# LIM kinase1 regulates mitotic centrosome integrity via its activity on dynein light intermediate chains

**DOI:** 10.1098/rsob.170202

**Published:** 2018-06-20

**Authors:** Sirong Ou, Mei-Hua Tan, Ting Weng, HoiYeung Li, Cheng-Gee Koh

**Affiliations:** 1 School of Biological Sciences, College of Science, Nanyang Technological University, 60 Nanyang Drive, 637551, Singapore; 2 Mechanobiology Institute, National University of Singapore, T-Lab, #05-01, 5A Engineering Drive 1, 117411, Singapore

**Keywords:** LIM kinase, centrosome integrity, dynein light intermediate chains, multi-polar spindle

## Abstract

Abnormal centrosome number and function have been implicated in tumour development. LIM kinase1 (LIMK1), a regulator of actin cytoskeleton dynamics, is found to localize at the mitotic centrosome. However, its role at the centrosome is not fully explored. Here, we report that LIMK1 depletion resulted in multi-polar spindles and defocusing of centrosomes, implicating its involvement in the regulation of mitotic centrosome integrity. LIMK1 could influence centrosome integrity by modulating centrosomal protein localization at the spindle pole. Interestingly, dynein light intermediate chains (LICs) are able to rescue the defects observed in LIMK1-depleted cells. We found that LICs are potential novel interacting partners and substrates of LIMK1 and that LIMK1 phosphorylation regulates cytoplasmic dynein function in centrosomal protein transport, which in turn impacts mitotic spindle pole integrity.

## 1. Introduction

The centrosome is the primary microtubule organizing centre (MTOC) in mammalian cells, and is involved in regulating cell motility, adhesion, polarity and mitotic spindle formation. The formation of an effective bipolar mitotic spindle is crucial to ensure faithful chromosome separation during cell division. Abnormal centrosome structure, function and number result in improper spindle formation, which can potentially lead to chromosome instability and tumourigenesis [1–3].

During mitosis, the centrosomes duplicate, mature and separate from each other to form a bipolar spindle. Centrosome maturation begins at G2/M phase transition, where several proteins are recruited to the pericentriolar material (PCM) to increase its capacity to organize, nucleate and anchor microtubules during mitosis. The maturation process is regulated by multiple kinases, including cyclin-dependent kinase 1 (CDK1), Aurora kinase A (AurkA) and Polo-like kinase 1 (PLK1) [4,5].

Several actin-associated proteins have been shown to localize at the mitotic spindle, suggesting their involvement in regulating spindle functions [6]. LIM kinase 1 (LIMK1) belongs to the LIM motif containing protein kinase (LIMK) family and regulates actin cytoskeleton dynamics through phosphorylation of cofilin [7,8]. CDK1 is reported to phosphorylate and activate LIMK1 during mitosis [9,10]. Phosphorylated LIMK1 then localizes at centrosomes during the M phase [11]. Although earlier findings have identified LIMK1 in regulation of spindle orientation and cytokinesis, the exact role of this kinase at the mitotic centrosome has not been fully explored [12–14].

LIMK1 and AurkA are reported to function together for proper spindle formation [15]. RNAi-mediated depletion of LIMK1 results in abnormal spindle morphology [15]. Interestingly, AurkA is reported to be a kinase as well as a substrate of LIMK1 [15]. LIMK1 and AurkA may form a positive feedback loop during mitosis for proper mitotic spindle formation.

The primary motivation of this study is to determine the functional links between LIMK1 and cell cycle control. Our observations that silencing LIMK1 leads to defective spindle organization and centrosome integrity further confirm such links. Here, we identify dynein light intermediate chains as the downstream mediators of LIMK1 in the maintenance of centrosome integrity.

## 2. Results

### 2.1. Silencing LIM kinase1 leads to centrosome defocusing and multi-polarity

**Figure 1.**
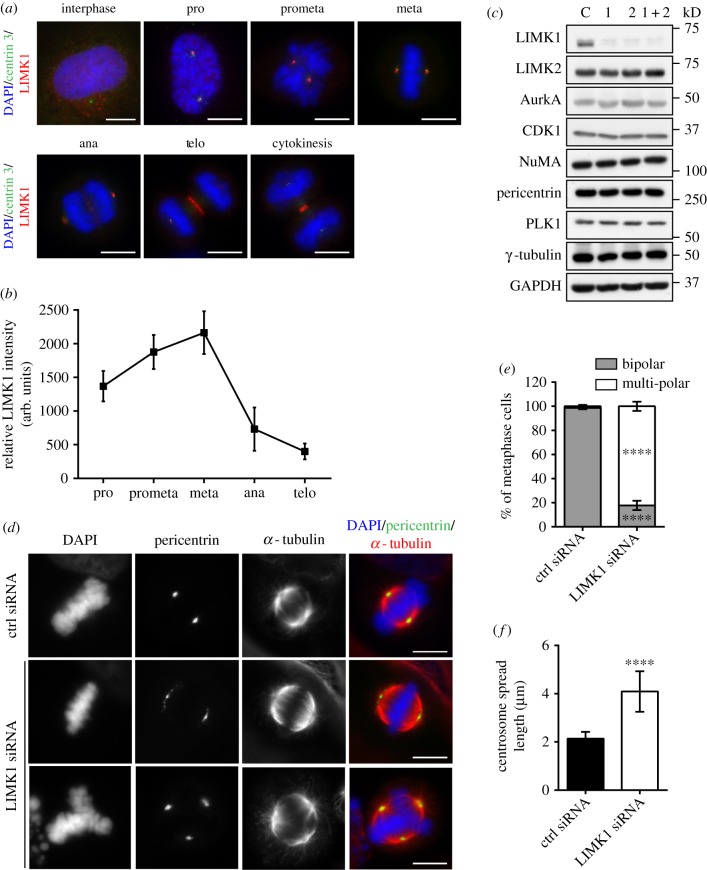
Silencing LIMK1 leads to multi-polar spindles. (*a*) HeLa cells were immuno-stained with anti-LIMK1 (red), anti-centrin 3 (green) and DAPI (blue) to visualize the location of LIMK1 at various phases of the cell cycle. Scale bar, 10 µm. (*b*) The relative intensity of LIMK1 immuno-stained at different stages of the cell cycle was quantified. The calculated intensity was normalized against the area of the selected foci and the mean LIMK1 fluorescence intensities were plotted. For each stage of M phase, 300 cells from three independent repeats were included for the analysis. The error bars represent standard deviation. arb. units, arbitrary units. (*c*) HeLa cells were transfected with two siRNAs targeting LIMK1 and a control siRNA for 48 h and synchronized with nocodazole. Protein cell lysates collected were run on SDS-PAGE and subjected to western analysis with the respective antibodies. C, control siRNA; 1, LIMK1 siRNA1; 2, LIMK1 siRNA2. (*d*) HeLa cells were transfected with control or LIMK1 siRNA for 48 h. The cells were immuno-stained with anti-pericentrin (green) and anti-α-tubulin (red) to visualize the centrosome and the mitotic spindle, respectively. Scale bar, 10 µm. (*e*) Cells were treated as described in (*d*) and the numbers of metaphase cells displaying bipolar and multi-polar spindles were counted. The mean proportion of metaphase cells displaying the respective phenotypes was calculated and plotted. The experiment was performed in triplicate; *n* = 300. The error bars represent standard deviation. ****p ≤ 0.0001, Student's *t*-test. (*f*) Cells were treated as described in (*d*) and the metaphase centrosome spread length was measured as described in Material and methods. The mean centrosome spread length was calculated and plotted. Experiment was performed in triplicate; *n* = 300. The error bars represent standard deviation. ****p ≤ 0.0001, Student's *t*-test.

As CDK1 is crucial for coordinating mitosis and can phosphorylate LIMK1, we hypothesized that LIMK1 might play functional roles during M phase. We first examined the localization pattern of LIMK1. LIMK1 was observed to localize close to the centrioles from prophase to metaphase (figure 1*a*). The localization is lost when the cells are treated with LIMK1 siRNA (electronic supplementary material, figure S1). To determine the changes in LIMK1 accumulation at the centrosome across mitosis, the intensity of LIMK1 at the foci was calculated (electronic supplementary material, figure S2). LIMK1 accumulation at the centrosome was at its maximum and reached a plateau at pro-metaphase and metaphase (figure 1*b*). After metaphase, LIMK1 localization at the centrosome gradually declined and was almost completely lost during telophase. This loss of LIMK1 from the centrosome coincided with the appearance of the kinase at the mid-body (figure 1*a* and electronic supplementary material, figure S3).

The localization of LIMK1 suggests possible function at the centrosome during mitosis. To investigate further, we designed siRNAs targeting two different sequences of the 3′-untranslated regions (3′-UTR) of LIMK1 mRNA. Both siRNAs significantly reduced the endogenous LIMK1 protein level, but not that of LIMK2 (figure 1*c*). The LIMK1 siRNAs also did not affect either the levels of other kinases which are regulators of the centrosomes or the levels of PCM materials (figure 1*c*). HeLa cells transfected with LIMK1 or control siRNA were immuno-stained with anti-pericentrin (centrosome marker) and anti-α-tubulin (mitotic spindle). Metaphase cells were identified through the arrangement of the chromosomes. We observed that approximately 97% of control cells formed bipolar spindles and the centrosomes were focused into two compact foci (figure 1*d,e*). By contrast, the proportion of metaphase cells forming bipolar spindles dropped to approximately 18.0% in LIMK1-depleted cells. Interestingly, most of the LIMK1-depleted cells (approx. 82.0%) formed multi-polar spindles, whereas the proportion of such cells in the control was very low (approx. 1.3%) (figure 1*d*,*e*). Astral and kinetochore microtubules were observed to radiate from each spindle pole, suggesting functional centrosomes. In addition to multi-polarity, the centrosomal material of LIMK1-depleted cells appeared to be diffused around the spindle poles (centrosome defocusing), which is consistent with our previous report [16]. Centrosome defocusing defects were quantified by measuring the centrosome spread length. The mean centrosome spread length was 2.14 µm in control cells. By contrast, the mean centrosome spread length of LIMK1 siRNA-treated cells (4.07 µm) was significantly longer (figure 1*f*). These studies imply that LIMK1 is important for the regulation of centrosome integrity during mitosis and consequently for the formation of a proper bipolar spindle.

### 2.2. Multi-polarity defects in LIM kinase1-depleted cells are due to pericentriolar material fragmentation

**Figure 2.**
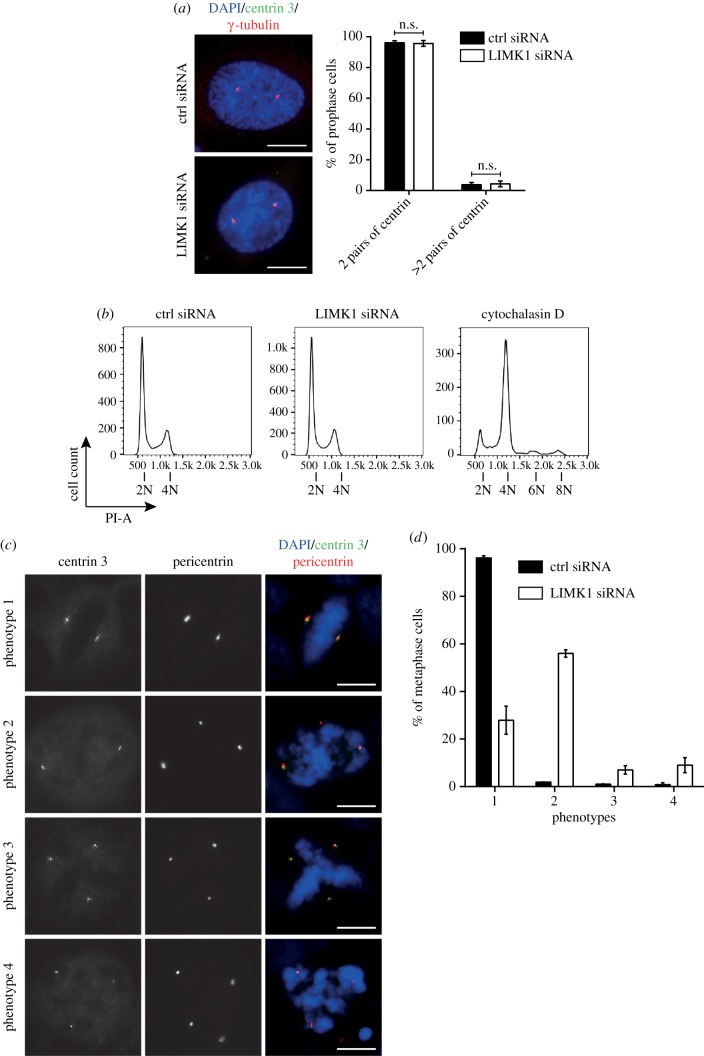
The multi-polar spindle defects induced by LIMK1 knockdown are due to the fragmentation of pericentriolar material (PCM). (*a*) HeLa cells were transfected with control and LIMK1 siRNAs. Immuno-staining was done using anti-centrin 3 (green) and anti-γ-tubulin (red) to visualize the centrioles and pericentriolar material, respectively. DAPI stains the nuclear DNA (blue). Scale bar, 10 µm. The percentages of cells with two pairs or more than two pairs of centrin were counted. There appears to be no difference between control and LIMK1 siRNA-transfected cells. n.s., *p* > 0.05. (*b*) FACS analysis. No obvious difference was observed between the DNA FACS profiles of LIMK1-knockdown and control. On the other hand, cells treated with cytochalasin D (control) showed extra peaks at 6N and 8N due to cytokinesis defects. (*c*) HeLa cells were transfected with either control or LIMK1 siRNA for 48 h. Transfected cells were then immuno-stained with anti-centrin 3 (green) and anti-pericentrin (red) antibodies, and DAPI (blue). Representative images of each phenotype are presented. Scale bar, 10 µm. (*d*) LIMK1 depletion leads to PCM fragmentation. The mean proportion of metaphase cells displaying the different phenotypes was scored in both control and LIMK1 siRNA-treated cells. Error bars represent the s.d. (*n* = 300). The experiment was performed in triplicate.

Previous studies have shown that centriole over-duplication and cytokinesis failure can lead to multi-polar spindle and chromosome instability [17]. We next investigated whether LIMK1 depletion led to cytokinesis failure or centriole over-duplication. The process of centrosome duplication is completed by early prophase [18]. To quantify the number of centriole pairs, HeLa cells transfected with either control or LIMK1 siRNA were immuno-stained with anti-γ-tubulin and anti-centrin 3 to visualize centrosome and centriole pairs and the number of centrin 3 pairs within the centrosomal foci per cell in early prophase cells was used for the quantification. From our observations of cells at early prophase, the number of centrin pairs per cell in LIMK1-depleted cells was not significantly different from that of control cells, suggesting that LIMK1 is not involved in the centriole duplication process (figure 2*a*).

In addition to the abnormal number of centrosomes, cytokinesis failure can lead to aneuploidy and multi-nucleated cells. Cells containing an abnormal number of chromosomes are reflected in the appearance of abnormal DNA content. However, the DNA fluorescence-activated cell sorting (FACS) profiles of LIMK1-depleted cells were similar to those treated with control siRNA (figure 2*b* and electronic supplementary material, figure S4b,c). In addition, LIMK1 depletion did not result in a significant increase in the number of multi-nucleated cells. Unlike LIMK1-knockdown cells, cells treated with cytochalasin D to disrupt the actin cytoskeleton showed cytokinesis defects and multi-nucleated cells (figure 2*b*). We also did not observe obvious apoptosis in LIMK1-knockdown cells (electronic supplementary material, figure S4a). These findings suggest that the multi-polarity observed in LIMK1-depleted cells was not due to cytokinesis failure.

Aberrant PCM fragmentation and/or centriole splitting have been reported to result in multi-polar spindle formation [19]. Therefore, we decided to investigate if LIMK1 depletion would result in PCM or centriole fragmentation in mitosis. LIMK1 and control siRNA-treated HeLa cells were immuno-stained with PCM protein (pericentrin) and centriole (centrin 3) markers. We classified the mitotic spindle pole morphology into four categories (figure 2*c*): Phenotype 1—cell forming two spindle poles and each pole containing a pair of centrioles (normal bipolar spindle); Phenotype 2—cell forming extra mitotic spindle poles but only two of the spindle poles contain a pair of centrioles; Phenotype 3—cell forming extra mitotic spindles with all the spindle poles containing centrioles; and Phenotype 4—cells containing extra mitotic spindle poles and centrioles but not all the spindle poles contain centrioles. If the majority of mitotic cells display Phenotype 2, it would suggest that the multi-polar spindle is due to PCM fragmentation. By contrast, Phenotypes 3 or 4 would suggest that multi-polar spindle formation is due to pre-mature centriole fragmentation, leading to pre-mature initiation of centriole duplication and formation of extra spindle poles.

In control siRNA-treated cells, about 96.2% of metaphase cells formed bipolar spindle (Phenotype 1) and only small percentages of control metaphase cells displayed Phenotypes 2, 3 or 4 (figure 2*c*,*d*). By contrast, only about 27.9% of LIMK1-depleted mitotic cells displayed Phenotype 1 (figure 2*c*,*d*). Consistent with earlier observations, LIMK1-depleted cells that formed bipolar spindle displayed centrosome defocusing defect. About 56.0% of LIMK1-depleted mitotic cells contained extra PCM foci with only two of them containing a pair of centriole, suggesting that these cells display Phenotype 2 (figure 2*c*,*d*). A very small percentage of LIMK1 siRNA-treated cells displayed Phenotypes 3 and 4 (7.0% and 9%, respectively) (figure 2*c*,*d*). We confirmed these observations using γ-tubulin (another PCM marker) and centrin 3 staining (electronic supplementary material, figure S5).

Taken together, our data suggest that the majority of LIMK1-depleted cells form multi-polar spindle due to PCM fragmentation during mitosis. Although pre-mature centriole fragmentation contributes to the formation of multi-polar spindle, it is not the major contributing factor in LIMK1-depleted cells.

### 2.3. LIM kinase1 depletion results in lower pericentriolar material protein accumulation at spindle poles

**Figure 3.**
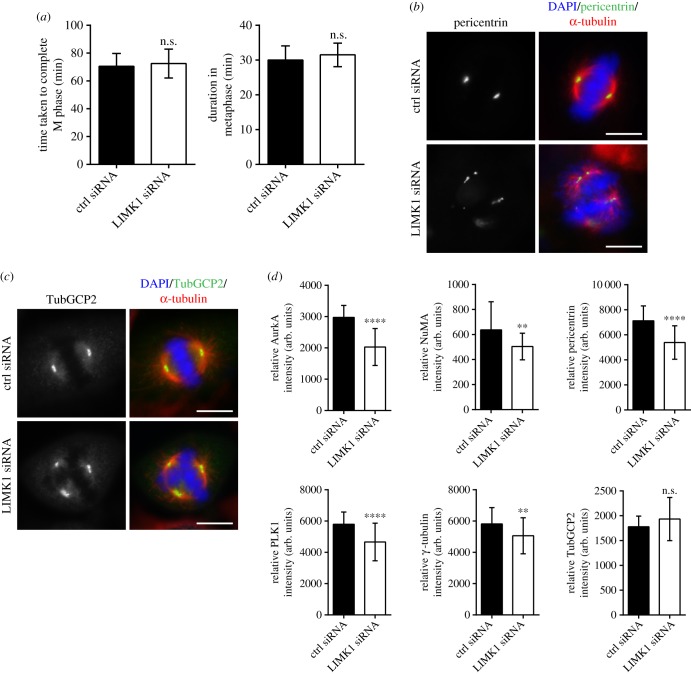
LIMK1 knockdown resulted in less protein accumulation at the centrosome. (*a*) The time taken for LIMK1 siRNA-transfected cells to go through M phase of the cell cycle was monitored by live-cell imaging. No obvious differences were observed between control and LIMK1 siRNA-transfected cells. (*b*) HeLa cells were transfected with control and LIMK1 siRNA. The cells were fixed and immuno-stained with anti-pericentrin (green), anti-α-tubulin (red) and for DNA (DAPI, blue). Scale bar, 10 µm. (*c*) HeLa cells were transfected with control and LIMK1 siRNA. The cells were fixed and immuno-stained with anti-TubGCP2 (green), anti-α-tubulin (red) and for DNA (DAPI, blue). Scale bar, 10 µm. (*d*) HeLa cells were transfected with control and LIMK1 siRNA. The cells were fixed and immuno-stained with the respective antibodies. The fluorescence intensities of AurkA, γ-tubulin, NuMA, pericentrin, PLK1 and TubGCP2 were measured and calculated as described in Material and methods. The calculated intensity was normalized against the area of the selected foci and plotted. Experiment was performed in triplicate; *n* = 300. The error bars represent standard deviation. arb. units, arbitrary units. ****p ≤ 0.0001; **p ≤ 0.01; n.s., *p* > 0.05.

Prolonged mitosis or metaphase–anaphase transition can result in excessive PCM protein accumulation at spindle poles, which can lead to cohesion fatigue, resulting in PCM fragmentation. Live cell imaging revealed that the average time taken for LIMK1-depleted cells to complete mitosis did not deviate significantly from the average time taken for the control cells (figure 3*a* and electronic supplementary material, figure S6). In addition, the metaphase–anaphase transition of LIMK1- and control-siRNAs treated cells was not significantly different (figure 3*a*). Thus, LIMK1 depletion did not affect mitotic progression.

Decreasing centrosomal accumulation of AurkA, nuclear mitotic apparatus protein (NuMA), pericentrin, PLK1, tubulin γ complex-associated protein 2 (TubGCP2) and γ-tubulin can compromise centrosome structural integrity, leading to PCM fragmentation. Therefore, we proceeded to measure the PCM protein accumulation at the spindle pole. The fluorescence intensities of the above-mentioned proteins at spindle poles were measured to quantify their accumulation at the centrosomes. We observed a significant decrease in the fluorescence intensities of AurkA, NuMA, pericentrin, PLK1 and γ-tubulin at spindle poles in LIMK1-depleted cells, (figure 3*b–d* and electronic supplementary material, figure S7a–d). Immunoblotting of centrosome isolated from LIMK1-treated cells also showed similar results (electronic supplementary material, figure S7e). Interestingly, the fluorescence intensity of TubGCP2 at metaphase centrosome was not significantly reduced in LIMK1 siRNA-treated cells, compared to the control cells (figure 3*c*,*d*). Our analysis suggested a role for LIMK1 in the regulation of PCM protein centrosomal accumulation and maintenance of centrosome structural integrity.

### 2.4. Active LIM kinase1 is required for the maintenance of proper centrosome and spindle organization

**Figure 4.**
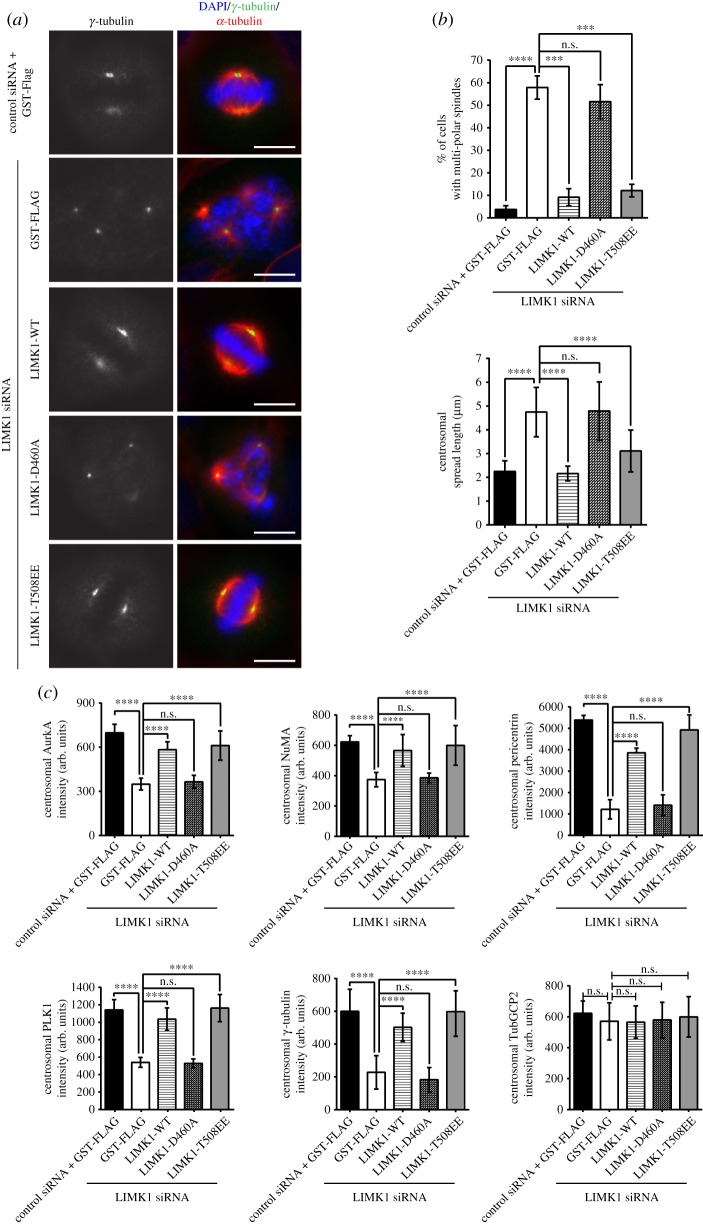
Active LIMK1 is required for the maintenance of proper centrosomal and spindle organization. (*a*) HeLa cells were transfected with the respective combination of control siRNA with GST-FLAG, LIMK1 siRNA and various LIMK1 constructs (LIMK1-WT, wild-type LIMK1; LIMK1-D460A, inactive LIMK1; LIMK1-T508EE, active LIMK1) for 48 h. Transfected cells were then harvested for immuno-fluorescence staining with anti-γ-tubulin (green) and anti-α-tubulin (red) antibodies to visualize mitotic centrosome and spindle, respectively. Mitotic chromosomes were stained with DAPI (blue). Scale bar, 10 µm. (*b*) The percentage of metaphase cells showing multi-polar phenotype was counted. The experiment was performed in triplicate; *n* = 300. The error bars represent standard deviation. The mitotic centrosome spread length was measured as described in Material and methods. The mean metaphase centrosome spread length was calculated and plotted. The experiment was performed in triplicate; *n* = 300. For both plots, the error bars represent standard deviation. ****p ≤ 0.0001; ***p ≤ 0.001; n.s., *p* > 0.05. (*c*) The fluorescence intensities of AurkA, NuMA, pericentrin, PLK1, γ-tubulin and TubGCP2 at the mitotic centrosome were measured and calculated as described in Material and methods. The calculated intensity was normalized against the area of the selected foci and plotted. Experiment was performed in triplicate; *n* = 300. The error bars represent standard deviation. arb. units, arbitrary units. ****p ≤ 0.0001; ***p ≤ 0.001; n.s., *p* > 0.05.

Earlier studies show that LIMK1 is hyper-activated during mitosis and its kinase activity is involved in regulating mitosis [9,10]. These earlier findings led us to hypothesize that the kinase activity of LIMK1 could be crucial for regulating centrosome integrity. To test this hypothesis, we generated and introduced kinase-dead LIMK1 mutant (LIMK1-D460A) or active LIMK1 mutant (LIMK1-T508EE) [20] into LIMK1-depleted cells (see electronic supplementary material, figure S8a for transfection efficiency). We found that when LIMK1-WT or LIMK1-T508EE is co-transfected with LIMK1 siRNA, there are fewer cells with multi-polar spindles (figure 4*a*,*b*). In addition, the centrosome spread length was also reduced (figure 4*b*). Inactive LIMK1 (D460A) was not able to rescue LIMK1-knockdown phenotypes. Expression of LIMK1-WT or LIMK1-T508EE also restores the levels of some centrosomal proteins (figure 4*c*).

### 2.5. LIC1 and 2 function downstream of LIM kinase1 in regulating centrosome integrity

Our earlier results suggest that LIMK1 depletion negatively affects AurkA, γ-tubulin, NuMA, pericentrin and PLK1 at mitotic centrosome. However, accumulation of TubGCP2 at the centrosome was not affected (figure 3*d*). Interestingly, all the centrosomal proteins, except for TubGCP2, were reported cargoes of cytoplasmic dynein 1 [21–25]. Cytoplasmic dynein 1 light intermediate chain 1 (LIC1) and 2 (LIC2) are two of the multiple subunits belonging to cytoplasmic dynein 1 motor complex. LIC1 and LIC2 define cargo specificity of dynein [26–29]. In addition, both LIC1 and LIC2 are reported to localize to the mitotic spindle and regulate key M phase processes [29–31]. Therefore, we hypothesized that LIC1/2 might function downstream of LIMK1 to regulate the transportation of centrosomal proteins, which in turn help to maintain the integrity of mitotic centrosome.

**Figure 5.**
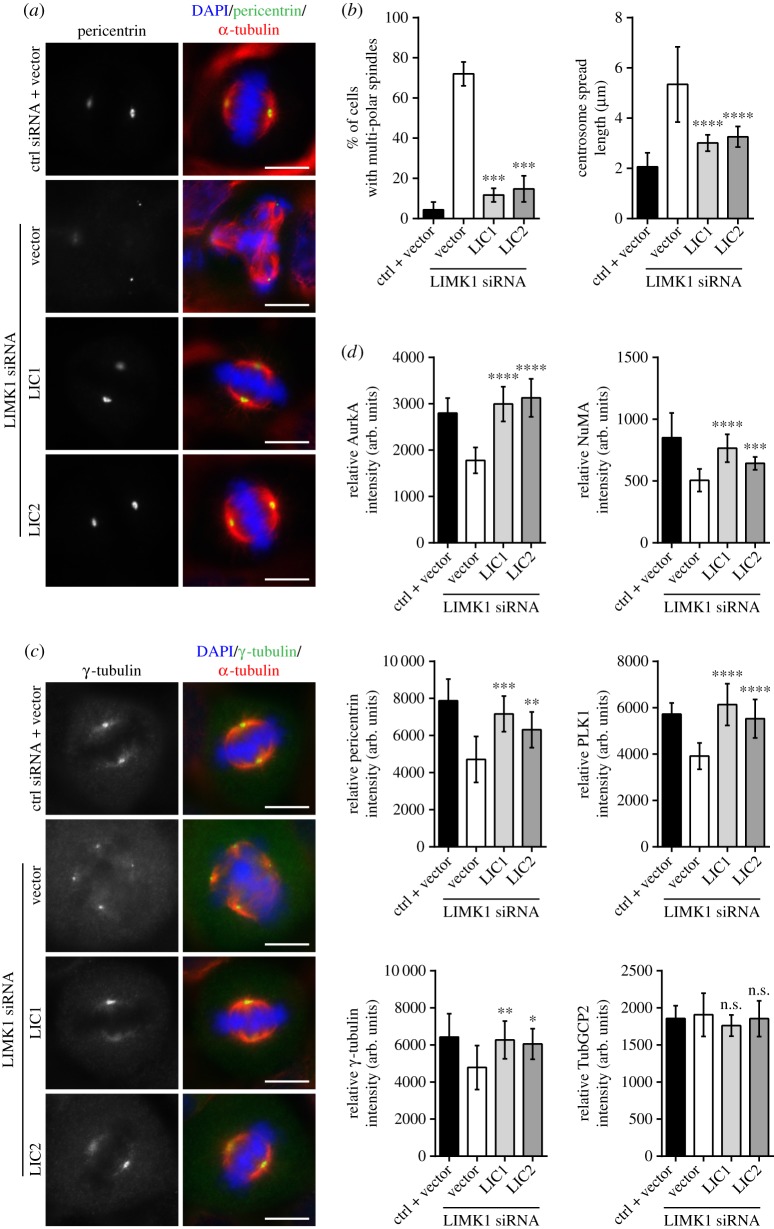
Introducing LIC1and LIC2 into LIMK1-depleted cells rescued the mitotic defects. (*a*) HeLa cells were transfected with the respective combination of siRNA and LIC constructs for 48 h. Treated cells were then fixed for immuno-fluorescence staining with anti-pericentrin (green) and anti-α-tubulin (red) antibodies to visualize centrosome and mitotic spindle, respectively. Mitotic chromosomes were stained with DAPI (blue). Representative images are presented. Scale bar, 10 µm. (*b*) The percentage of metaphase cells showing multi-polar phenotype was counted. The experiment was performed in triplicate; *n* = 300. The error bars represent standard deviation. The mitotic centrosome spread length was measured as described in Material and methods. The mean metaphase centrosome spread length was calculated and plotted. The experiment was performed in triplicate; *n* = 300. For both plots, the error bars represent standard deviation. ****p ≤ 0.0001; ***p ≤ 0.001. (*c*) Same as in (*a*) except that anti-γ-tubulin (green) was used to visualized the centrosomes. (*d*) The fluorescence intensities of AurkA, NuMA, pericentrin, PLK1, γ-tubulin and TubGCP2 at the mitotic centrosome were measured and calculated as described in Material and methods. The calculated intensity was normalized against the area of the selected foci and plotted. Experiment was performed in triplicate; *n* = 300. The error bars represent standard deviation. arb. units, arbitrary units. ****p ≤ 0.0001; ***p ≤ 0.001, **p ≤ 0.01, *p ≤ 0.05, n.s., *p* > 0.05.

We introduced LIC1 and LIC2 constructs into LIMK1 siRNA-treated cells and monitored mitotic centrosome defects. Cells co-transfected with control siRNA and GST-FLAG construct served as controls for comparison. We observed that the proportion of cells displaying multi-polar spindle decreased when LIC1 and LIC2 were introduced into LIMK1-depleted cells (11.7% and 14.8%, respectively) (figure 5*a*,*b* and electronic supplementary material, figure S8b). This decrease was significant when compared to cells co-transfected with LIMK1 siRNA and GST-FLAG (72.0% cells show multi-polar spindle; *p* ≤ 0.001) (figure 5*a*,*b*). Besides reducing the number of cells displaying multi-polar spindle, introduction of either LIC1 or LIC2 was able to reduce the mean metaphase centrosome spread length (figure 5*b*). These observations suggest that LIC1 and LIC2 could rescue the defects in LIMK1-depleted cells and could potentially be novel substrates functioning downstream of LIMK1 in regulating mitotic centrosome integrity.

Next, we investigated if LIC1 and LIC2 are able to restore the centrosomal protein levels at the spindle poles in LIMK1-depleted cells. We introduced either LIC1 or LIC2 into LIMK1 siRNA-treated cells. The fluorescence intensities of the centrosomal proteins were calculated as described in the previous section (figure 5*c* (only γ-tubulin staining was shown)). Cells co-transfected with control siRNA and GST-FLAG construct served as controls for comparison. Similar to earlier experiments, we focused our efforts on AurkA, γ-tubulin, NuMA, pericentrin, PLK1 and TubGCP2 at the mitotic spindle poles. Our measurement showed that the fluorescence intensities of these proteins at spindle poles, except for TubGCP2, were restored to levels similar to those of the control (figure 5*d*). These results suggest that both LICs could potentially function downstream of LIMK1 in regulating the transportation of centrosomal protein.

### 2.6. LIC1 and LIC2 depletion results in similar defects as LIM kinase1 knockdown

Earlier we showed that introducing LIC1 and LIC2 into LIMK1-depleted cells reduced metaphase centrosome defocusing and cells forming multi-polar spindles. In addition, we demonstrated that both LICs restored the centrosomal protein levels in LIMK1-knockdown cells. These data raised the possibility that LIC1/2 might function downstream of LIMK1 in regulating centrosome integrity. Therefore, we decided to determine if silencing endogenous LIC1 and LIC2 could lead to similar phenotypes as LIMK1 knockdown.

**Figure 6.**
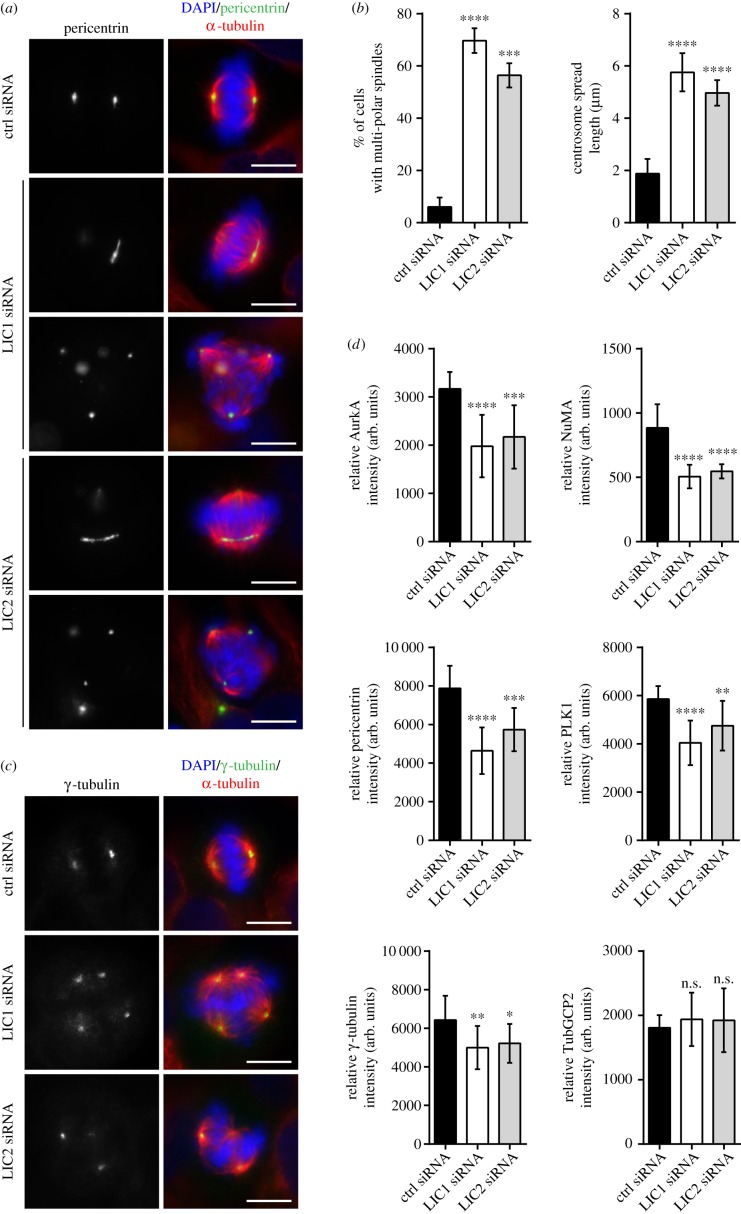
Knocking down LIC1 or LIC2 resulted in multi-polar spindles. (*a*) HeLa cells were transfected with the respective siRNAs for 48 h. Treated cells were then processed for immuno-fluorescence staining with anti-pericentrin (green) and anti-α-tubulin (red) antibodies to visualize centrosome and mitotic spindle, respectively. Mitotic chromosomes were stained with DAPI (blue). Representative images are presented. Scale bar, 10 µm. (*b*) The percentage of metaphase cells showing multi-polar phenotype was counted. The experiment was performed in triplicate; *n* = 300. The error bars represent standard deviation. The mitotic centrosome spread length was measured as described in Material and methods. The mean metaphase centrosome spread length was calculated and plotted. The experiment was performed in triplicate; *n* = 300. For both plots, the error bars represent standard deviation. ****p ≤ 0.0001; ***p ≤ 0.001. (*c*) Same as in (*a*) except that anti-γ-tubulin (green) was used to visualize the centrosomes. Scale bar, 10 µm. (*d*) The fluorescence intensities of AurkA, NuMA, pericentrin, PLK1, γ-tubulin and TubGCP2 at the mitotic centrosome were measured and calculated as described in Material and methods. The calculated intensity was normalized against the area of the selected foci and plotted. Experiment was performed in triplicate; *n* = 300. The error bars represent standard deviation. arb. units, arbitrary units. ****p ≤ 0.0001; ***p ≤ 0.001, **p ≤ 0.01, *p ≤ 0.05, n.s., *p* > 0.05.

We knocked down LIC1 and LIC2 in HeLa cells (electronic supplementary material, figure S8c) and monitored the resulting centrosome defects. We observed that about only 6.0% of the control cells forms multi-polar spindles. By contrast, about 70.0% of LIC1 knockdown cells and 56.0% of LIC2 knockdown cells formed multi-polar spindles (figure 6*a*,*b*).

We next examined the mean metaphase centrosome spread length (figure 6*b*). Similar to our earlier findings, the mean metaphase centrosome spread length was about 1.9 μm for control cells. When cells were treated with either LIC1 siRNA (5.0 µm) or LIC2 siRNA (5.8 µm), the mean metaphase centrosome spread length was significantly increased. These observations suggest that depleting either one of the LICs leads to centrosome defocusing.

Lastly, we investigated if depleting LIC1 and LIC2 would result in lower centrosomal protein accumulation at the mitotic spindle poles. HeLa cells transfected with control, LIC1 or LIC2 siRNA were monitored as described earlier (figure 6*c*). We observed that the fluorescence intensities of AurkA, γ-tubulin, NuMA, pericentrin and PLK1 at the mitotic spindle poles were reduced in either LIC1 or LIC2 knockdown cells (figure 6*d*). The fluorescence intensity of TubGCP2 serves as a control, because it is not a reported cargo of cytoplasmic dynein 1. Our observations suggest that LIC depletion has a negative effect on centrosomal protein accumulation.

Taken together, our data show that similar to LIMK1 knockdown, depletion of either LIC1 or LIC2 results in centrosome defocusing and multi-polar spindle formation. Treating cells with either LIC1 or LIC2 siRNA also reduces the accumulation of proteins at the mitotic centrosome.

### 2.7. LIC1 and LIC2 are novel interacting partners and substrates of LIM kinase1

**Figure 7.**
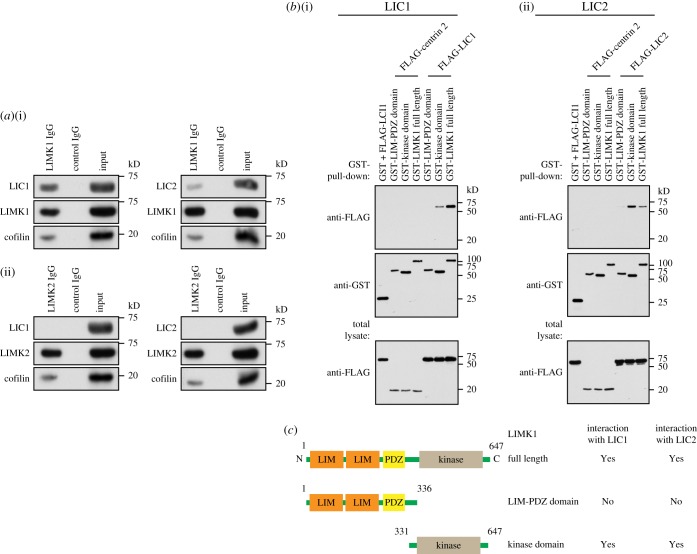
LIC1 and LIC2 interact with LIMK1. (*a*) HeLa cells were first synchronized to M phase by treating the cells with nocodazole for 16 h. After synchronization, cell lysates were collected and incubated with either anti-LIMK1 (i) or anti-LIMK2 (ii) for endogenous immuno-precipitation assays. The immuno-precipitated proteins were then subjected to western blot analysis and probed for endogenous LIC1, LIC2 and the respective LIMKs. Non-immunized rabbit IgG antibodies were included as negative control for the immuno-precipitation assays. LIC1 and LIC2 are found to co-precipitate with LIMK1 but not LIMK2. (*b*) LIC1 and LIC2 interact with the kinase domain of LIMK1. HEK 293 cells were transfected with the respective combinations of LIMK1 and LIC1 or LIC2 constructs. Twenty-four hours after transfection, cell lysates were harvested and subjected to GST-pull-down. Total lysates and GST-pull-down fractions were subjected to western blot analysis. Lysate and GST-pull-down fractions were probed with FLAG and GST antibodies to detect LIC1, LIC2 and LIMK1 constructs, respectively. FLAG-centrin 2 was used as a negative control. HEK293 cells were used as they show higher efficiency for transfection experiments. (*c*) Summary of interaction between LIC1 and LIC2, and various LIMK1 constructs. N and C represent N-terminus and C-terminus, respectively. The numbers above the constructs represent the amino acid residue numbers.

As LIC1 and LIC2 were able to rescue the defects observed in LIMK1-knockdown cells, they could potentially function downstream of LIMK1. We next performed endogenous protein immuno-precipitation assay to determine if LIMK1 interacts with LICs. HeLa cells were first synchronized to M phase using nocodazole, cell lysates were collected and incubated with LIMK1-IgG antibodies for the immuno-precipitation assay. LIMK2-IgG antibodies were included as negative control. We found that LIC1 and LIC2 co-immuno-precipitated with LIMK1 (figure 7*a*). LIMK2, a closely related protein kinase of LIMK1, did not co-immuno-precipitate with LIC1 or LIC2. These data suggest that LIMK1, but not LIMK2, interacts with both LICs during M phase. Interestingly, we also observed LIC1/2 interaction with LIMK1 in asynchronized cells (electronic supplementary material, figure S9a).

To narrow down the domain on LIMK1 which could potentially interact with LICs, we generated two GST-tagged LIMK1 constructs containing either the LIM-PDZ or the kinase domain (figure 7*c*). We found that LIC1 and LIC2 co-precipitated with LIMK1 kinase domain, but not with LIM-PDZ (figure 7*b*,*c*). Consistently, we observed less LIC interaction with the kinase-dead mutant LIMK1-D460A (electronic supplementary material, figure S9b).

**Figure 8.**
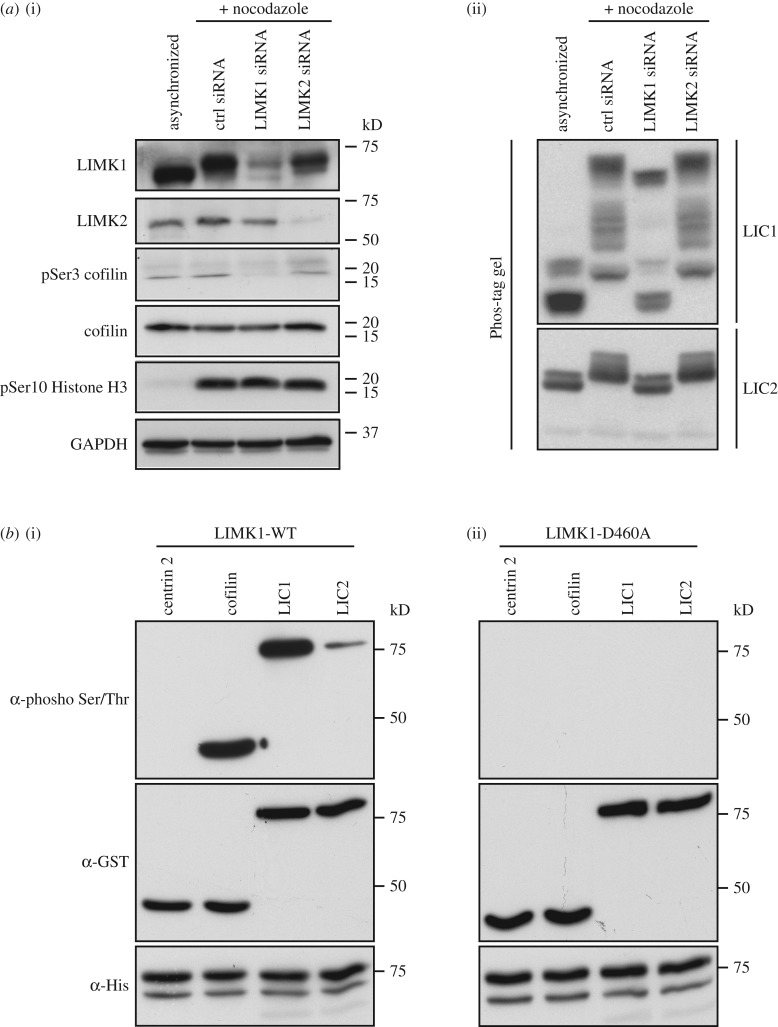
LIC1 and LIC2 are substrates of LIMK1. (*a*) LIMK1 depletion affects LIC phosphorylation. HeLa cells were treated with the respective siRNAs and synchronized at M phase with nocodazole. Synchronized cell lysates were then collected and the efficacies of the respective siRNAs were investigated using normal SDS-PAGE (i), phospho-Histone H3 level serves as M-phase marker. Endogenous GAPDH level serves as the loading control. The phosphorylation profiles of LIC1 and LIC2 were investigated using Phos-tag PAGE (ii). Both acrylamide gels were then subjected to western blot analysis. (*b*) Bacterial expressed GST-centrin 2, GST-cofilin, GST-LIC1 and GST-LIC2 proteins were incubated with His-LIMK1-WT or the inactive His-LIMK1-D460A. The reaction mixes were separated by SDS-PAGE and analysed by western blot using anti-phospho-Ser/Thr antibodies. LIMK1-WT could phosphorylate cofilin and LIC1 and LIC2 but not centrin 2. Inactive LIMK1-D460A could not phosphorylate any of the GST-tagged proteins.

The LICs could be potential substrates of LIMK1 during M phase. We proceeded to examine the phosphorylation profiles of LIC1 and LIC2 in LIMK1 siRNA-treated cells using Phos-tag polyacrylamide gel electrophoresis (Phos-tag PAGE). HeLa cells were first treated with either LIMK1 or control siRNAs and synchronized to M phase. Cell lysates were then harvested and subjected to Phos-Tag PAGE analysis. LIMK2 siRNAs were included as additional controls. From the Phos-tag PAGE analysis, we observed that several slower migrating LIC1 and LIC2 bands were present in M phase, but not in interphase cell lysates (figure 8*a*). This observation suggests that some residues or combination of residues on LICs are specifically phosphorylated during mitosis. When cells were transfected with LIMK2 siRNAs, the mitotic phosphorylation profiles of both LICs were similar to those of cells treated with control siRNA (figure 8*a*). By contrast, the mitotic phosphorylation profiles of both LICs were altered when the cells were transfected with LIMK1 siRNA, indicating that LIMK1 depletion affects the phosphorylation of LIC1 and LIC2. To confirm our hypothesis that LIMK1 is an upstream kinase of LICs, we performed an *in vitro* kinase assay. Both LIC1 and LIC2 were phosphorylated in the presence of wild type, but not kinase-dead LIMK1 (figure 8*b*). As LIMK1 is also reported to phosphorylate tyrosine [32], we determined if any tyrosine residues in LIC1 and LIC2 were phosphorylated by LIMK1 (electronic supplementary material, figure S9c). Indeed, LIMK1 was able to phosphorylate LIC1 and LIC2 at tyrosine residues. Together with the results obtained from GST-pull-down assay and Phos-Tag PAGE analysis, it is reasonable to speculate that LICs are potential substrates of LIMK1.

As LICs could be potential LIMK1 substrates, we investigated how LIMK1 might affect dynein motor function. We tracked the trafficking of PLK1, which is a cargo of the dynein motor, in cells co-transfected with GFP-PLK1 together with vector, active or inactive LIMK1 constructs. We found that when the kinase-dead LIMK1-D460A was overexpressed, the speed of PLK1 trafficking was altered compared to that of control (electronic supplementary material, figure S10), suggesting that the activity of LIMK1 could affect dynein motor function.

## 3. Discussion

Abnormal centrosome structure, function and number can result in improper spindle formation, which could potentially lead to chromosome instability and tumourigenesis. Although earlier findings showed that LIMK1 regulates spindle orientation and cytokinesis, the exact role of this kinase in mitosis is not explored. Our current study suggests that LIMK1 is important for maintaining mitotic centrosome integrity by regulating PCM protein accumulation. Based on our experimental findings, we proposed the following working model: during G2/M-phase transition, LIMK1 is phosphorylated and activated by an M-phase kinase, such as CDK1. Activated LIMK1 then phosphorylates LICs, which in turn regulate the interaction between dynein and PCM proteins or the trafficking of PCM proteins, thus modulating the transportation of PCM proteins to the mitotic spindle poles. These PCM proteins are required for the maintenance and structural integrity of the mitotic centrosome.

As cytokinesis defect could potentially lead to abnormal centrosome number in subsequent cell division, we initially hypothesized that the multi-polar spindle observed in LIMK1-depleted cells could be due to cytokinesis failure. Surprisingly, our data did not support this. We did not observe abnormal DNA content in LIMK1-knockdown cells (figure 2*b* and electronic supplementary material, figure S4b). In addition, LIMK1 siRNA treatment did not significantly increase the number of multi-nucleated interphase cells (electronic supplementary material, figure S4c). Collectively, these data suggest that LIMK1 depletion does not lead to cytokinesis failure. It is not immediately clear why our findings contradict earlier reports, which suggest involvement of LIMK1 in cytokinesis. However, other studies have also shown that transient expression of LIMK1 prolongs mitosis timing, but cells exit mitosis without cytokinesis defects [33]. The observations that cells with LIMK1 knockdown are capable of completing cytokinesis might be due to possible redundant function of LIMK2 as LIMK2 is localized to the midzone microtubules during anaphase to telophase [34]. It is also possible that the activity of LIMK1 is not required for cytokinesis as LIMK1 is hyperphosphorylated at the onset of M phase and gradually becomes inactivated during telophase and cytokinesis [9,10]. Although we observe multi-polar spindles in LIMK1-knockdown cells, these extra spindle poles may eventually coalescence/cluster to become bipolar spindles before anaphase onset especially in cancer cells [35]. We also reported more cells exhibiting a multi-polar spindle in LIMK1-knockdown situations than LIMK2-knockdown situations [16]. In addition, our Phos-tag SDS-PAGE analysis demonstrated that depletion of LIMK1, but not of LIMK2, affected the phosphorylation of LICs (figure 8), suggesting specific functions and requirement for LIMK1.

It has been proposed that prolonging metaphase–anaphase transition leads to excessive accumulation of centrosomal protein at the spindle poles, which results in unstable spindle poles. This instability, in turn, leads to centrosome fragmentation and multi-polarity [36]. Although we observed multi-polar spindles in LIMK1-depleted cells, our data do not support this hypothesis. Instead, we found that there were lower amounts of centrosomal proteins at the spindle poles (figure 3*d*).

PLK1 phosphorylates and recruits centrosomal protein Kizuna (Kiz) to the centrosome [37]. Kiz then acts as a ‘linking bridge' to bind other PCM proteins, thus preventing mitotic spindle pole fragmentation. NuMA has also been shown to be important for maintaining the bipolar status of the mitotic spindle [38]. Immuno-depletion of NuMA from *Xenopus* egg extracts disrupts microtubule anchorage to the spindle pole and prevents the formation of a focused spindle pole [23]. The reduced accumulation of NuMA and PLK1 could potentially explain the defocused centrosomes and multi-polar spindles observed in LIMK1-knockdown cells.

In this study, we found that LIMK1 interacts with LIC1/2. However, we cannot conclude that LIMK1–LIC interaction is dependent on microtubules. This is because we synchronized the cells at M phase using 50 nM nocodazole. It has been reported that nanomolar concentrations of nocodazole can block cells in mitosis without disassembling the microtubule network but by reducing the microtubule dynamics instead [39,40]. At this concentration, nocodazole treatment leads to improper spindle structure and DNA alignment due to reduction in microtubule growth and shortening velocities instead of depolymerizing the existing microtubule network.

Dynein and its adaptors and binding proteins play important roles in different stages of the cell cycle [41]. In addition, depletion of LICs has been implicated in centriole cohesion and multi-spindle formation [42]. As phosphorylation of dynein motor subunits has been shown to regulate cargo–motor interactions and trafficking [43,44], we speculate that LIMK1-mediated phosphorylation of LIC1 and LIC2 could affect loading or unloading of the PCM protein cargoes. In addition, LIMK1-mediated phosphorylation of LIC1 and LIC2 might affect the trafficking of PCM proteins as suggested by our data (electronic supplementary material, figure S10). Interestingly, overexpression of LIC1/2 could rescue the phenotypes elicited by silencing LIMK1. One possible explanation is that under the situation of overexpression, other kinases might be able to phosphorylate the abundant LICs. Cdc2 kinase has been reported to phosphorylate LIC and regulate dynein transport [43]. Alternatively, the residual amount of the remaining LIMK1 is capable of phosphorylating a portion of the overexpressed LICs. It appears that LIMK1-mediated phosphorylation, together with other post-translational modifications, could enhance the accumulation of PCM proteins onto the mitotic centrosome during centrosome maturation. In conclusion, our current work demonstrated a new role for LIMK1 in maintaining centrosomal integrity and the bipolar spindle. Active LIMK1 is required for regulating dynein function through phosphorylation of LICs.

## 4. Material and methods

### 4.1. Cell culture, drug treatment and transfections

HeLa cells were cultured in minimum essential medium (Sigma Aldrich) supplemented with 10% fetal bovine serum, 2.2 g l^−1^ sodium bicarbonate and 2 mM l-glutamine. The cells were maintained in a humidified incubator at 37°C in 5% CO_2_. Cells were synchronized to G2/M phase of the cell cycle by treating with 50 ng ml^−1^ nocodazole (Sigma Aldrich) for 16 h. Cells were subjected to cytochalasin D (0.5 mg ml^−1^) for 16 h to disrupt the actin cytoskeleton.

All transfections were performed using Lipofectamine 2000 according to the manufacturer's protocol. For all transfections, cells were seeded onto 35-mm dishes 24 h prior to transfection. For siRNA transfection, 150 pmol of Stealth siRNA (Invitrogen) was mixed with 3 µl Lipofectamine 2000 (Invitrogen) and incubated at room temperature for 20 min. The siRNA/Lipofectamine mixture was then added to and incubated with the cells for 48 h. For plasmid transfection, 1 μg of plasmid was mixed with 3 μl Lipofectamine-2000 and incubated at room temperature for 20 min. If two or more plasmid constructs were transfected into cells, the amounts of plasmids were adjusted such that the total amount of plasmids transfected would be 1 μg. The DNA/Lipofectamine mixture was then added to and incubated with the cells for 24 or 48 h as described. For rescue experiments, cells were first transfected with siRNA for 24 h and subsequently transfected with respective plasmid constructs. Twenty-four hours after plasmid transfection, cells were processed for subsequent experiments.

### 4.2. Antibodies and siRNA

Antibodies used in this project include antibodies against pericentrin (Abcam), LIMK1 (Cell Signaling), LIMK2 (Cell Signaling), AurkA (Abcam), centrin 3 (Santa Cruz Biotechnology), LIC1 (Abcam), LIC2 (Abcam), PLK1 (Abcam), α-tubulin (Sigma Aldrich), γ-tubulin (Sigma Aldrich), TubGCP2 (Sigma Aldrich) and NuMa (Cell Signaling).

siRNA sequences are as follows.

LIC1 sense: AGUGCUUCUUCAGAUGGACUAAAUU;

LIC2 sense: CACUUUCUAACAGGGUGGAGCAAAU;

LIMK1siRNA1 sense: CCUCUUGCUUCUCCUUGCAUGAGCU;

LIMK1 siRNA2 sense: CAACAGGUAUCGAGGACUCUCCAAA;

Luciferase sense: ACAUCACGUACGCGGAAUACUUCGA.

Cells were incubated with 150 pmol of stealth siRNA (Invitrogen)/Lipofectamine 2000 (Invitrogen) mixture for 48 h, according to the manufacturer's protocol.

### 4.3. Immuno-fluorescence microscopy

Cells were fixed and permeabilized in methanol at −20°C for 5 min, rehydrated with 1× PBS for 30 min and blocked with 4% bovine serum albumin (BSA) for 1 h. Cells were then incubated with the respective primary antibodies, washed with 0.1% Triton X/1× PBS, incubated with the appropriate secondary antibodies, and washed again with 0.1% Triton X/1× PBS. Immuno-stained samples were then counter-stained with DAPI contained in a mounting medium (Vectashield). All primary and secondary antibodies were diluted in 0.2% Triton X/1× PBS at a ratio of 1 : 500 and 1 : 1000, respectively. All immuno-stained samples were examined using the Axio Observer D1 microscope (Zeiss) equipped with either an EC Plan-Neofluar 40×/1.30 or a Plan-Apochromat 63×/1.40 oil immersion objective lens (Zeiss). AxioVision software was used to capture images using a CoolSNAP HQ2 camera. Images were then analysed and processed using ImageJ software.

### 4.4. Time-lapse imaging

HeLa cells were transfected with GFP-H2B to visualize chromosomes and chromatin at different stages of the cell cycle. Time-lapse fluorescent microscopic analysis was performed on an Axio Observer D1 microscope (Zeiss) equipped with a Plan-Apochromat 63×/1.4 oil immersion objective lens (Zeiss), a sample heater (37°C) and a CO_2_ incubation chamber. Images of the cell were captured with a CoolSNAP HQ2 camera every 5 min controlled by AxioVision software through the whole mitotic phase.

### 4.5. Centrosome defocusing and centrosomal protein fluorescence intensity quantification

To quantify centrosome defocusing, the centrosome spread length was measured. HeLa cells were immuno-stained with anti-pericentrin antibodies to visualize the centrosome. Metaphase cells were selected for *z*-stack (0.5 µm intervals) microscopy to visualize all spindle poles in a cell. A line, parallel to the metaphase plate, was drawn on the pericentrin foci and the length of the line represented the centrosome spread length.

For centrosomal protein foci intensity quantifications, fixed cells were immuno-stained with the respective antibodies. Metaphase cells were imaged at fixed microscope camera exposure settings. The centrosomal protein foci were outlined and background-corrected integrated fluorescence intensity was calculated for all individual centrosomal protein foci. The calculated intensity was then normalized against the area of the centrosomal protein foci (electronic supplementary material, figure S2).

### 4.6. Western analyses

Cells were lysed in mammalian cell lysis buffer (25 mM Hepes, pH 7.5, 0.25 M NaCl, 1 mM MgCl_2_, 1 mM EGTA, 20 mM *p*-glycerol phosphate, 1 mM sodium vanadate, 10 mM NaF, 5% glycerol, 0.5% Triton X-100, 5 mM DTT, 10 mg ml^−1^ DNase I, 1× protease inhibitor cocktail (Roche), 1× phosphatase inhibitor cocktail (Roche)). Cell lysates were separated by SDS-PAGE using 10% or 8% polyacrylamide gels and transferred onto nitrocellulose or PVDF membranes. For Phos-tag SDS-PAGE, Mn^2+^-based Phos-tag SDS-PAGE resolving and stacking gels containing 20 µM Phos-tag and 0.1 mM MnCl_2_ were prepared according to the manufacturer's protocol (Wako Pure Chemical, Osaka, Japan). The membranes were blocked with 5% BSA and probed with one of the following antibodies: mouse monoclonal anti-actin (1 : 5000; Chemicon International), rabbit polyclonal anti-AurkA (1 : 1000; Abcam), mouse monoclonal anti-GAPDH (1 : 5000; Ambion), rabbit polyclonal anti-LIMK1 (1 : 1000; Cell Signaling Technology), rabbit polyclonal anti-LIMK2 (1 : 1000; Cell Signaling Technology), rabbit polyclonal anti-NuMA (1 : 1000; Cell Signaling Technology), rabbit polyclonal anti-pericentrin (1 : 2000; Abcam), rabbit monoclonal anti-PLK1 (1 : 1000; Abcam), rabbit polyclonal anti-TubGCP2 (1 : 1000; Sigma Aldrich), mouse monoclonal anti-α-tubulin (1 : 5000; Sigma Aldrich) and rabbit polyclonal anti-γ-tubulin (1 : 1000; Sigma Aldrich); horseradish peroxidase-conjugated anti-mouse (1 : 5000) or anti-rabbit (1 : 5000) antibodies (Dako Cytomation). ECL Plus Chemiluminescent Detection Kit (Amersham) was used for detection.

### 4.7. GST pull-down and immuno-precipitation

HEK 293 cells were transfected with GST-tagged constructs for 24 h. Cell lysates were harvested. Thirty micrograms of total lysates was kept to determine the expression of the respective constructs (input) and the remaining lysate was incubated with 50 μl of Glutathione Sepharose^®^ 4B slurry beads (Amersham Biosciences). Beads were washed thrice with mammalian cell lysis buffer. Bound proteins were recovered from beads by adding 50 μl of 2× SDS sample buffer and analysed by western blotting.

For immuno-precipitation assay, HeLa cells were lysed with mammalian cell lysis buffer and 30 µg of total lysates was kept to determine the endogenous protein levels (input). The remaining cell lysates were pre-cleared with irrelevant antibody slurry beads and incubated with antibodies to proteins of interest or control antibodies, which were immobilized on magnetic Protein A slurry beads (Merck Millipore). Beads were washed four times with mammalian cell lysis buffer. Bound proteins were recovered from beads by adding 2× SDS sample buffer. The supernatants recovered were analysed by western blotting.

### 4.8. Centrosome isolation

HeLa cells were synchronized with 0.2 µM nocodazole for 1 h at 37°C. Cells were sequentially washed with 1× TBS, 0.1× TBS and 8% sucrose (w/v) in 0.1× TBS, and later lysed in lysis buffer containing 0.1× TBS and 8% sucrose and left to stand for 10 min at 4°C. Crude centrosomes were isolated by 60% sucrose centrifugation at 25 000*g* for 15 min at 4°C. The crude centrosomal fraction (bottom 20%) was collected and subjected to a second round of sucrose step gradient (70% : 50% : 40%) centrifugation at 120 000*g* for 1 h, at 4°C. Separate fractions containing 0.5 ml were collected and analysed by SDS-PAGE.

### 4.9. *In vitro* kinase assay

The substrates were purified as GST-fusion proteins from *Escherichia coli*. The kinases were recovered using glutathione sepharose beads from COS-7 cells transfected with constructs encoding different mutants of GST-LIMK1. The *in vitro* kinase assay was performed by incubating different combinations of substrate and kinase in kinase buffer containing 50 mM Hepes, pH 7.5, 25 mM β-glycerol phosphate, 5 mM MgCl_2_, 5 mM MnCl_2_, 1 mM Na_3_VO_4_ and 500 μM ATP at 30°C for 45 min. The reaction mix was then assayed by SDS-PAGE and western blot analysis.

## Supplementary Material

Supplementary Materials

## Data accessibility

All the material concerning this work are/will be made publicly available and are already provided in this manuscript and supplemental figures. We did not generate datasets or codes for this study. Accepted manuscript and data will be uploaded to Nanyang Technological University repository.
