# Supplementary material for: LIM kinase1 regulates mitotic centrosome integrity via its activity on dynein light intermediate chains
Source: Open Biol. 2018 Jun 20;8(6):170202. doi: 10.1098/rsob.170202 (PMC6030115; doi:10.1098/rsob.170202)
Supplement: Supplementary Materials [file rsob170202supp1.pdf]

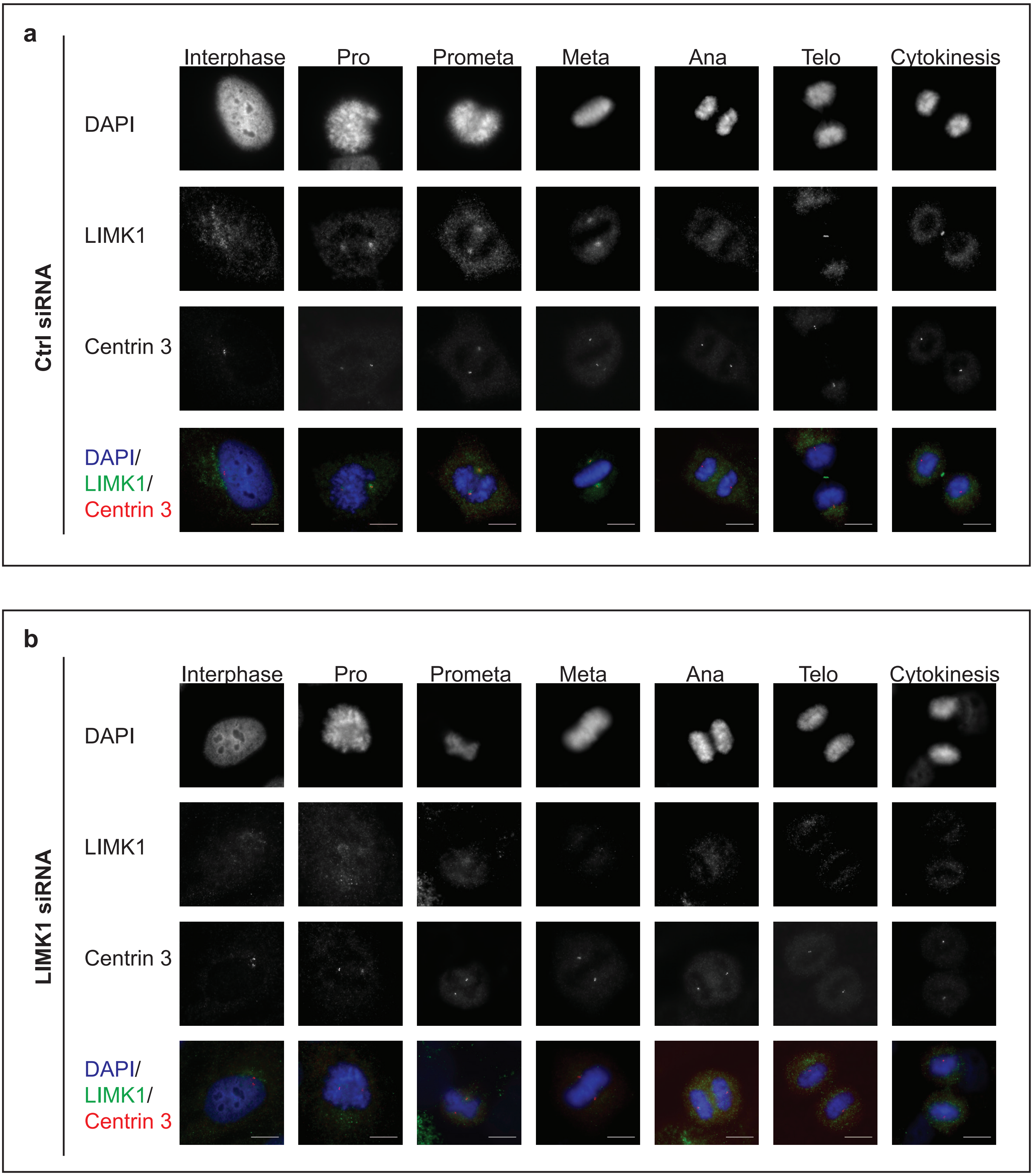

Supplementary Fig. S1. HeLa cells were transfected with **(a)** control siRNA, and **(b)** LIMK1 siRNA, for 48 hours. Immunostaining was done using anti-LIMK1 (green) and anti-centrin 3 (red). DAPI stains the nuclear DNA (blue). Scale bar: 10 μm.

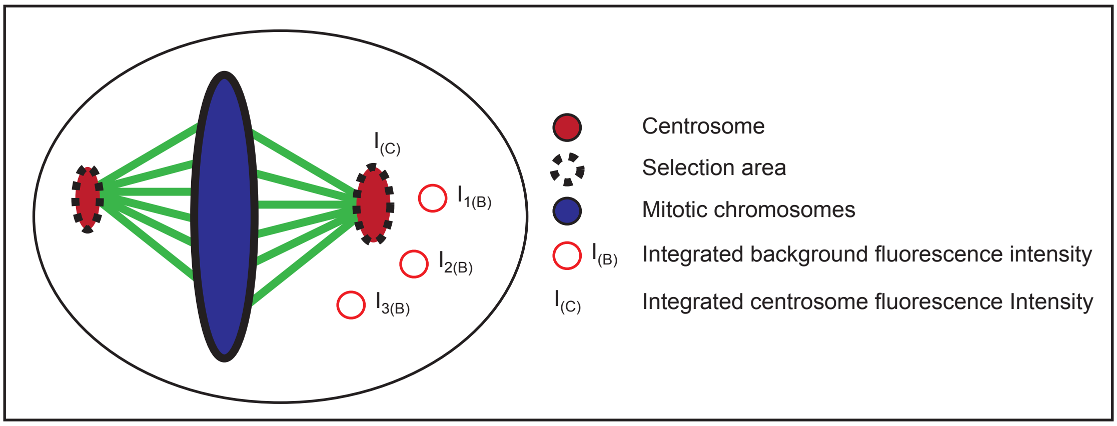

Supplementary Fig. S2. Schematic diagram of quantification of centrosomal-located LIMK1 fluorescence intensity. The centrosomal foci were delimited by outlining the area containing LIMK1 fluorescent intensity according to the staining of Centrin 3 and LIMK1. To calculate the fluorescence intensity of LIMK1 within the foci, three different non-centrosomal areas in the cell were chosen to get the average fluorescence intensity of the background ($A_{(B)}$) according to this formula: $A_{(B)} = I_{(B)}/S_{(B)}$, $I_{(B)}$ represents the fluorescence intensity measured, $S_{(B)}$ represents the total area of the background selected.

Centrosomal LIMK1 fluorescence intensity = $[\, I_{(C)} - A_{(B)} \times S_{(C)}] / S_{(C)}$. $I_{(C)}$ represents the fluorescence intensity measured, $S_{(C)}$ represents centrosomal area selected.

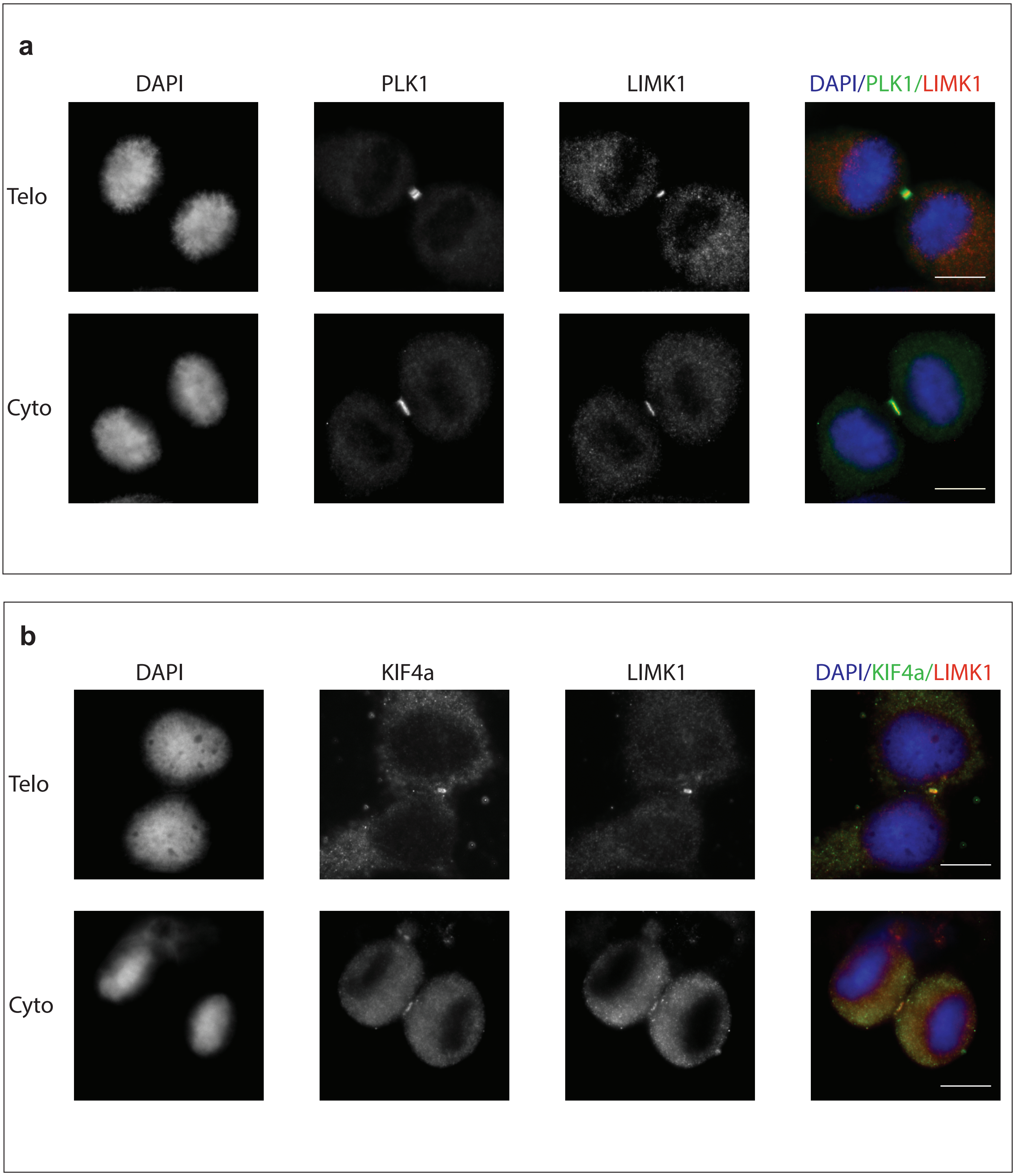

Supplementary Fig. S3. HeLa cells were immunostained with anti-LIMK1 (red) and co-stained with **(a)** anti-PLK1 (green) and **(b)** anti-KIF4a (green), which are reported to localized to the midbody. DAPI stains the nuclear DNA (blue). Scale bar: 10μm.

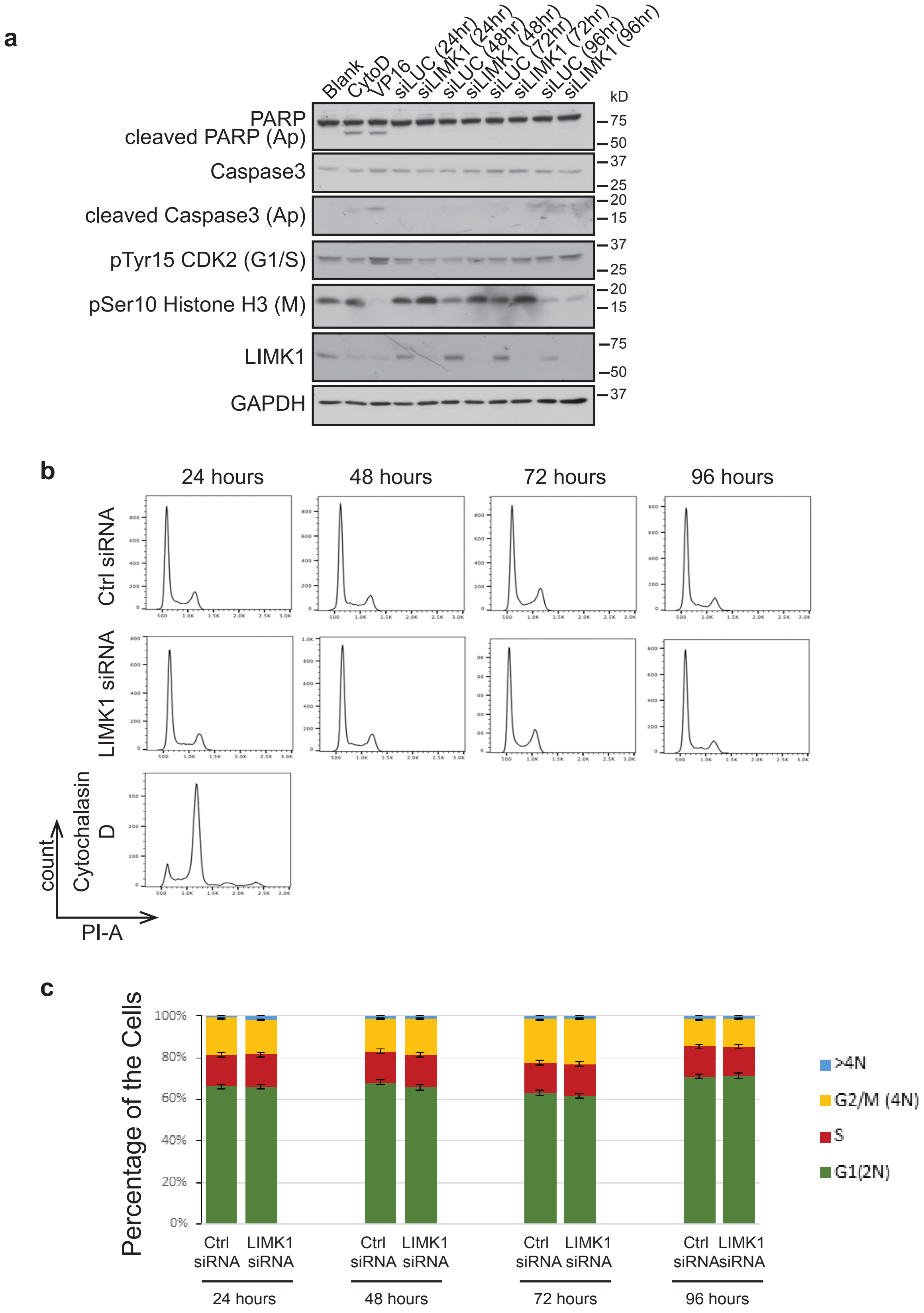

Supplementary Fig. S4. (a) HeLa cells were transfected with control- and LIMK1- siRNAs. Total cell lysates were harvested for Western blot analysis at different time points as indicated. No observable cleavage of PARP and Caspase-3 was detected in the control and LIMK-knock-down cells, indicating that transient LIMK1 knockdown did not result in obvious apoptosis. No significant difference was observed between the control and LIMK1- knockdown cells in terms of G1/S phase marker, phospho-CDK2 (Tyr15), levels. Cells with LIMK1 knockdown showed higher levels of M phase marker, phospho-Histone H3, than control cells. Ap represents apoptotic marker. Cells treated with VP-16 are positive control for apoptosis. Cells treated with Cytochalasin D are positive control for cytokinesis failure. **(b)** Control and LIMK1-knockdown cells were subjected to FACS at different time points as stated in (a) to investigate the DNA contents. Cells treated with Cytochalasin D were used as positive control for aneuploidy. The control and LIMK1 knockdown cells showed similar DNA profile. **(c)** Statistic analysis of (b) from three biological repeats.

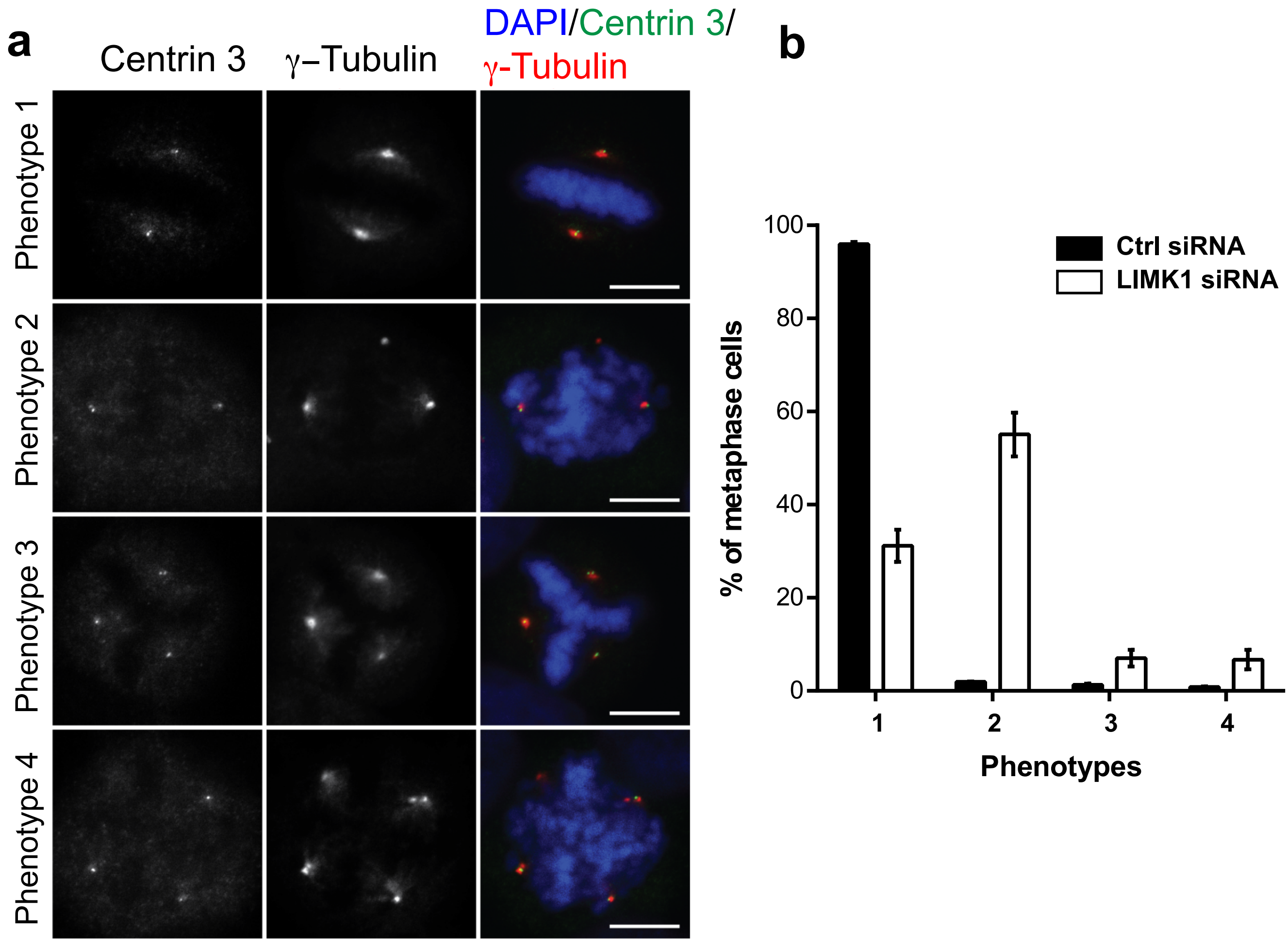

Supplementary Fig. S5. (a) HeLa cells were transfected with control- and LIMK1-siRNAs. Immunostaining was done using anti-centrin 3 (green) and anti-γtubulin (red) to visualize the centrioles and peri-centriolar material, respectively. DAPI stains the nuclear DNA (blue) Scale bar: 10 μm. **(b)** The mean proportion of metaphase cells displaying the different phenotype was scored in both control and LIMK1 siRNA treated cells. Error bars represent the s.d. (n = 300). The experiment was performed in triplicate.

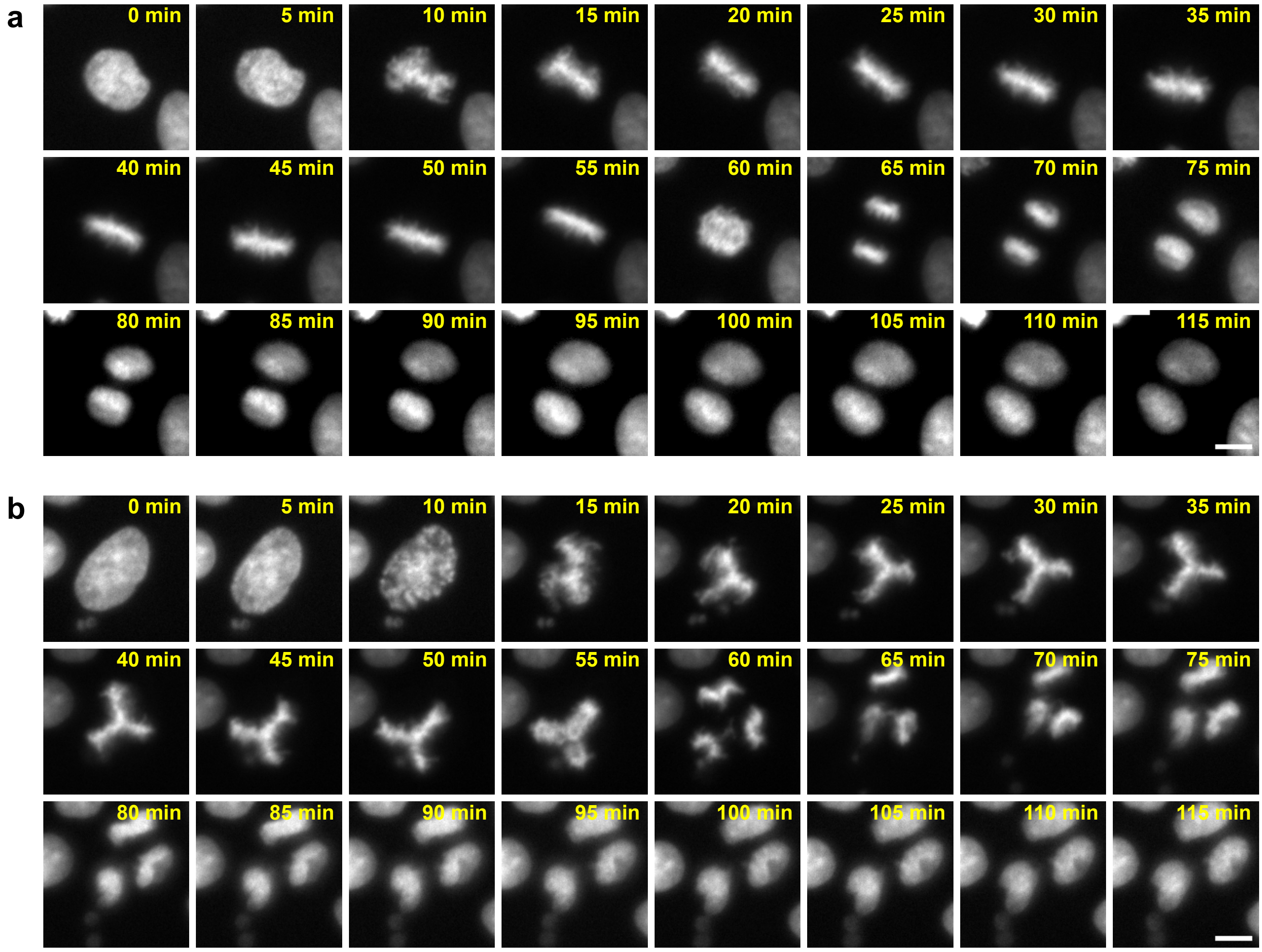

Supplementary Fig. S6. Live imaging time-lapse series of HeLa cells transfected with GFP-H2B together with **(a)** control siRNA or **(b)** LIMK1 siRNA. The duration of mitosis and metaphase appear similar for control and LIMK1 siRNA transfected cells. Scale Bar: 10μm.

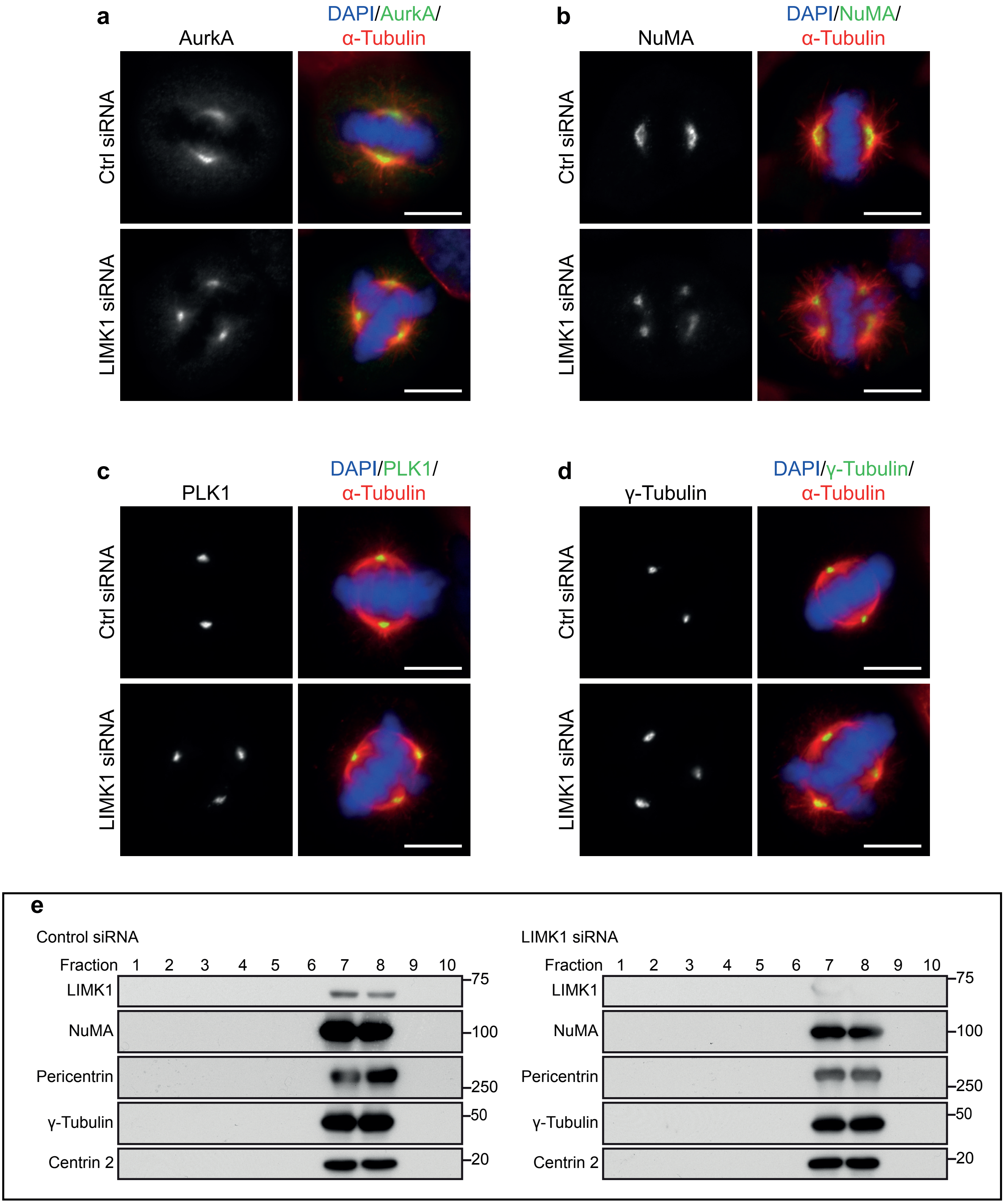

Supplementary Fig. S7. HeLa cells were transfected with control and LIMK1 siRNA. The cells were fixed and immunostained with the respective antibodies: **(a)** AurkA (green), **(b)** NuMa (green), **(c)** PLK1 (green), **(d)** γ-tubulin together with α-tubulin (red), DAPI (blue). Scale bar: 10 μm. **(e)** HeLa cells were transfected with control and LIMK1 siRNAs for 48 hours. The cell lysate was harvested and fractionated. The centrosomal fractions were subjected to SDS-PAGE and western analysis. Less NuMA, pericentrin, γ-tubulin were found in the centrosome of LIMK1 siRNA transfected cells. Centrin 2 served as the loading control.

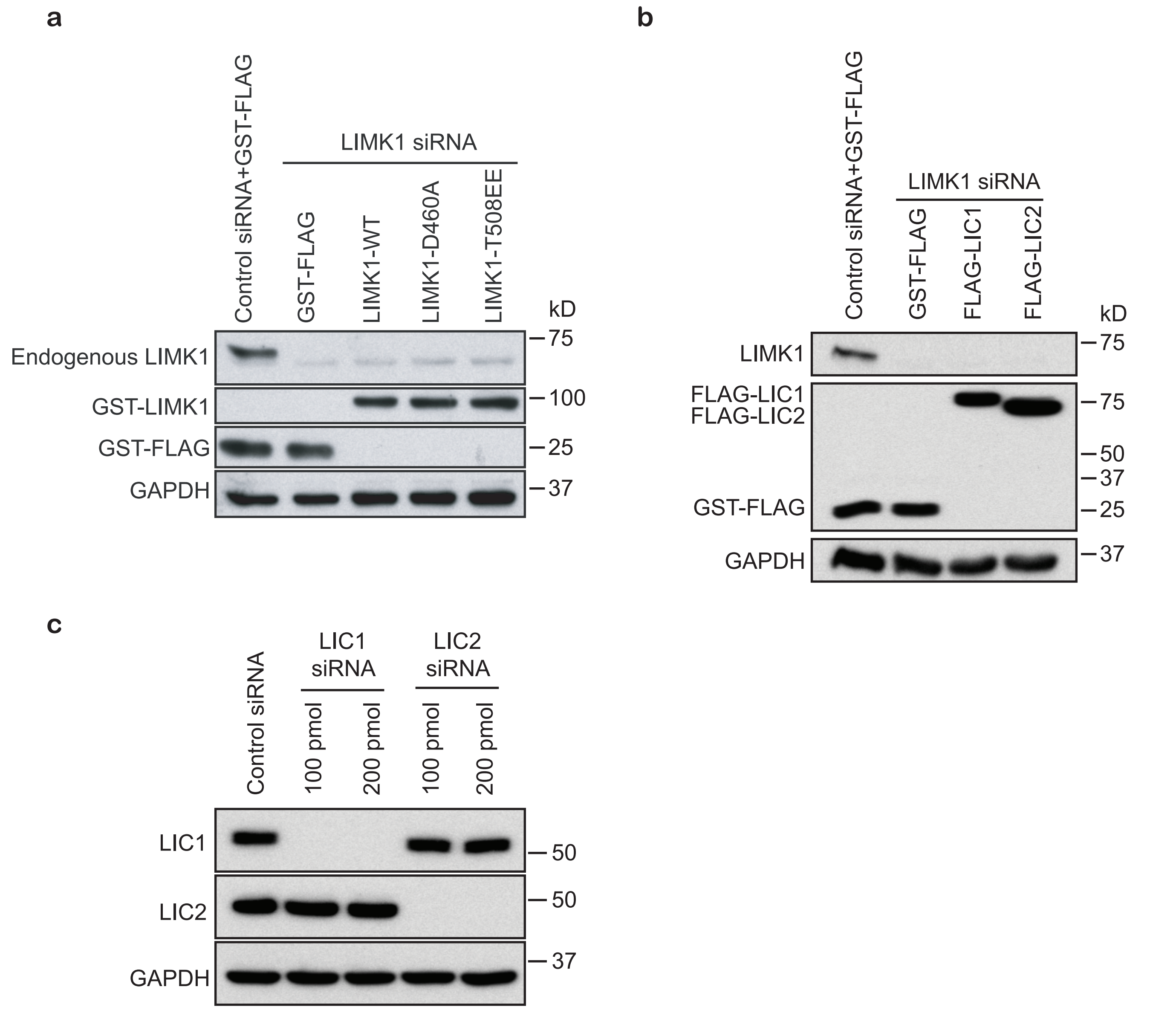

Supplementary Fig. S8. (a) HeLa cells were first transfected with control or LIMK1 siRNA for 24 hours and subsequently transfected with different constructs as indicated for another 24 hours. The expression levels of endogenous LIMK1 and the transfected GST-tagged proteins were determined using Western blot. **(b)** HeLa cells were first transfected with different siRNAs for 24 hours, followed by transfection of different FLAG-constructs as indicated. Total cell lysates were subjected to SDS-PAGE and Western blot analysis to determine the levels of LIMK1 and FLAG-tagged proteins. **(c)** HeLa cells were transfected with different amount of siRNAs targeting LIC1 and LIC2 to titrate the dosage of siRNAs needed to silence LICs. The levels of LICs were determined by Western blot analysis. In subsequent experiments involving LIC1 or LIC2 knockdown, 100 pmol of the respective siRNAs were used.

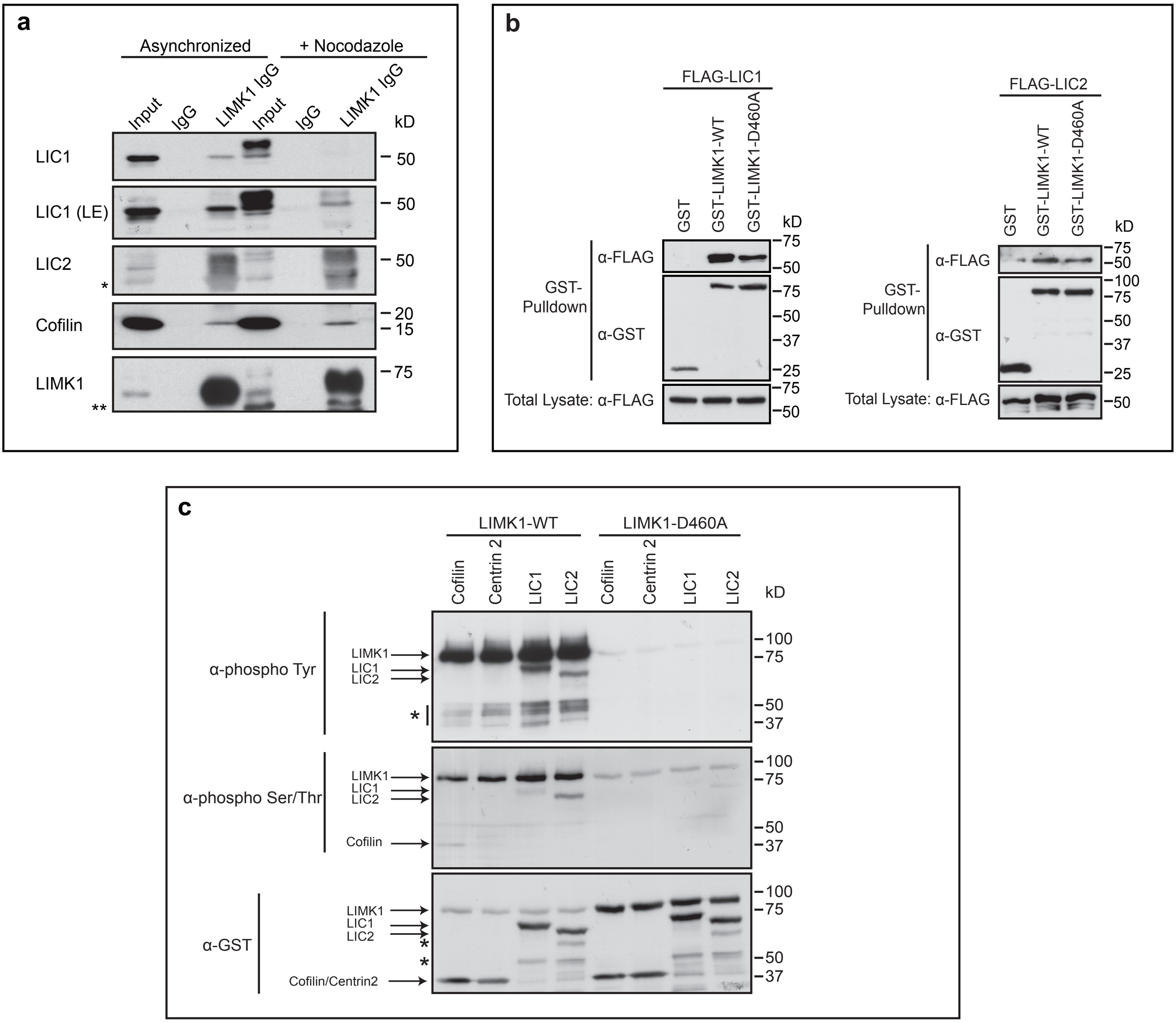

Supplementary Fig. S9. (a) LIC1 and 2 interact with LIMK1. HeLa cells were either untreated or synchronized to M-phase by treating the cells with nocodazole for 16 hours. Cell lysates were then collected and incubated with either random IgG or anti-LIMK1 antibodies for endogenous immuno-precipitation assays. The immunoprecipitated complex were then subjected to Western blot analysis and probed for endogenous LIC1, LIC2, and LIMK1. LIC1 and 2 are found to co-precipitate with LIMK1 in both asynchronized and M-phase cells. (LE): long exposure; *: non-specific band of LIC2 antibody; **: bands of LIC1 (residual from stripped blots). **(b)** The interactions of LIC1 (left panel) or LIC2 (right panel) with wildtype (LIMK1-WT) and kinase dead mutant of LIMK1 (LIMK-D460A) were determined. COS-7 cells were transfected with different combinations of plasmids. GST or GST-LIMK1 were recovered by GST pulldown and subjected to Western blot analysis. LICs associated with LIMK1 were detected with anti-FLAG antibody. LIMK1-D460A shows decreased interaction with LICs when compared with LIMK1-WT. **(c)** Bacterial expressed GST-cofilin, GST-centrin 2, GST-LIC1 and GST-LIC2 proteins were incubated with GST-LIMK1-WT or the inactive GST-LIMK1-D460A isolated from COS-7 cells transfected with the respective constructs. The reaction mixtures are incubated at 30 ^{o}C for 45 mins in kinase buffer containing ATP. The reaction mixtures were separated by SDS-PAGE and analysed by Western blot using anti-phospho-Tyr and anti-phospho-Ser/Thr antibodies. LIMK1-WT could phosphorylate cofilin and LIC1/2 but not centrin 2 at Ser/Thr residues. LIMK1-WT could phosphorylate LIC1/2 at Tyr residues. The inactive LIMK1-D460A could not phosphorylate any of the GST-tagged proteins. *: non-specific bands.

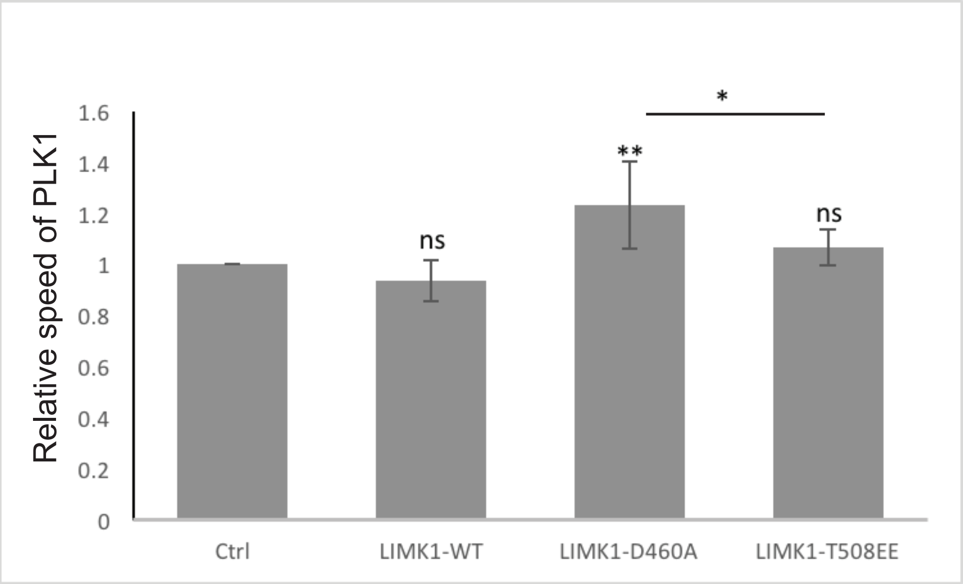

Supplementary Fig. S10. Relative speed of PLK1 in HeLa cells transfected with GFP-PLK1 together with mCherry (ctrl), mCherry-LIMK1-WT, mCherry-LIMK1-D460A and mcherry-LIMK1-T508EE for 24 hours. Transfected cells were subjected to live imaging at interval of 5 seconds for 5 minutes. The speed of each GFP-PLK1 was calculated using Trackmate, Image J. Relative speed was normalised against the ctrl setup. Experiment was performed in triplicates; n=300. The error bars represent standard deviation. ** represents $p \leq 0.01$; * represents $p \leq 0.05$; ns repesents $p \geq 0.05$.
